# Honey Bee Allatostatins Target Galanin/Somatostatin-Like Receptors and Modulate Learning: A Conserved Function?

**DOI:** 10.1371/journal.pone.0146248

**Published:** 2016-01-07

**Authors:** Elodie Urlacher, Laurent Soustelle, Marie-Laure Parmentier, Heleen Verlinden, Marie-Julie Gherardi, Daniel Fourmy, Alison R. Mercer, Jean-Marc Devaud, Isabelle Massou

**Affiliations:** 1 Department of Zoology, Dunedin, Otago, New Zealand; 2 Centre National de la Recherche Scientifique (CNRS), Centre de Recherches sur la Cognition Animale (UMR 5169), Toulouse, France; 3 Université de Toulouse, UPS Centre de Recherches sur la Cognition Animale (UMR 5169), Toulouse, France; 4 CNRS, UMR 5203, Institut de Génomique Fonctionnelle, Montpellier, France; 5 INSERM, U1191, Montpellier, France; 6 Université de Montpellier, UMR 5203, Montpellier, France; 7 Department of Animal Physiology and Neurobiology, Zoological Institute, KU Leuven, Leuven, Belgium; 8 EA 4552 Réceptorologie et ciblage thérapeutique en cancérologie, Université de Toulouse, UPS, Toulouse, France; University of Würzburg, GERMANY

## Abstract

Sequencing of the honeybee genome revealed many neuropeptides and putative neuropeptide receptors, yet functional characterization of these peptidic systems is scarce. In this study, we focus on allatostatins, which were first identified as inhibitors of juvenile hormone synthesis, but whose role in the adult honey bee (*Apis mellifera*) brain remains to be determined. We characterize the bee allatostatin system, represented by two families: allatostatin A (Apime-ASTA) and its receptor (Apime-ASTA-R); and C-type allatostatins (Apime-ASTC and Apime-ASTCC) and their common receptor (Apime-ASTC-R). Apime-ASTA-R and Apime-ASTC-R are the receptors in bees most closely related to vertebrate galanin and somatostatin receptors, respectively. We examine the functional properties of the two honeybee receptors and show that they are transcriptionally expressed in the adult brain, including in brain centers known to be important for learning and memory processes. Thus we investigated the effects of exogenously applied allatostatins on appetitive olfactory learning in the bee. Our results show that allatostatins modulate learning in this insect, and provide important insights into the evolution of somatostatin/allatostatin signaling.

## Introduction

Neuropeptide signaling is ubiquitous across animal phyla, and exerts a great variety of actions on animal physiology and behavior [1]. Through their actions as neuromodulators, neuropeptides play a central role in the coordinated regulation of an organism’s internal state [2] and in mammals, neuropeptidergic control of the motivational or emotional state has been shown in turn to have a significant impact on higher cognitive processes [3]. The actions of neuropeptides on learning and memory can be complex. In rodents for example, somatostatin modulates learning either positively or negatively, through the targeting of different receptors (reviewed in [3]). While there is evidence for state-dependent modulation of learning and memory in invertebrates, and particularly in insects [4–6], little is known about the involvement of neuromodulatory peptides: to our knowledge, short neuropeptide F (sNPF) is the only neuropeptide for which such an effect has been described so far, at least in *Drosophila melanogaster* [7]. However, the identification of large numbers of neuropeptides in insects, and the expression of many of these peptides in the brain, suggest that neuropeptide signaling is likely to contribute significantly to normal brain function in insects and possibly modulate cognition also in these animals [1,7–10].

We have chosen to investigate this possibility using the honey bee, *Apis mellifera*. The honey bee provides an interesting and tractable model that, from a number of different perspectives, is ideal for analyzing neuropeptide signaling and its impacts on insect cognition. The complex social organization of honey bee colonies demands a high degree of neural and behavioral plasticity, enabling individuals to respond appropriately to colony needs and to display higher-order cognitive processes that support activities such as the learning and recall of food-related cues. The honey bee has been used extensively in studies investigating the neural bases of learning and memory, and honey bee brain networks support highly efficient and surprisingly complex learning abilities [11–12]. The honey bee is therefore an ideal choice for addressing experimentally whether neuropeptides in insects modulate learning and memory processes.

More than one hundred neuropeptides have been identified from the honey bee genome [13], but less than 15 of these have been successfully detected in the honey bee brain [14]. Among these are several allatostatins [14], neuropeptides that inhibit the biosynthesis of juvenile hormone in the *corpora allata* of insects [15–18], can reduce food intake [19–22] and modulate foraging [23–24]. Like somatostatins in mammals, allatostatins were originally identified as regulators of developmental growth [15–17], but these peptides are present also in the adult nervous system and their role(s), within the adult brain in particular, remain unclear.

Neuropeptide nomenclature is subject to debate [25], especially for allatostatins. 'Allatostatin' refers to an inhibitory effect on the *corpora allata*, and as peptides with no structural homology from different insect species were identified to have such effect, they were sequentially categorized into three subfamilies of allatostatins (A, B, C [16]). Sequencing of numerous insect genomes allowed the identification of many putative allatostatins based on sequence similarity rather than biological activity (the peptides identified in this study are currently being investigated for their allatostatic activity). *Apis mellifera* A-type allatostatins (Apime-ASTAs) share the Y/FXFGL-NH2 consensus sequence [26–27], and include at least 5 peptides in the honey bee brain, the most abundant form in the brain being A4 (GRQPYSFGL-amide) [14,28]. While no gene encoding for B-type allatostatins has been identified in *Apis mellifera*, in bees as in several other insect species, two paralogue genes encode for C-type peptides [19,29]. However, these so-called C (SYWKQCAFNAVSCF-amide) and CC (GQAKGRVYWRCYFNAVTCF) allatostatins (Apime-ASTC and Apime-ASTCC) lack the consensus C-terminal PISCF sequence typical of the C subfamily [19,30], hence we chose to still call them allatostatins C instead of PISCF/AST as recommended by [25]. While the pattern of expression of Apime-ASTC and Apime-ASTCC has yet to be determined, Apime-ASTA is known to be widely expressed in the honey bee brain, including in antennal lobes and mushroom bodies [28], centers of the brain that are critical for learning and recall of olfactory memories (reviewed by [31]). However, the functional significance of allatostatins in these brain regions remains unknown. Two candidate receptors (GB19021 and GB20155) have been identified *in silico* as putative Apime-ASTA and Apime-ASTC receptors [9], but their expression in the bee brain and localization is yet to be determined.

To determine whether allatostatins modulate olfactory learning performance in bees, we have taken advantage of a well-established olfactory conditioning protocol that has been used extensively to investigate the cellular and molecular mechanisms that underlie appetitive learning and memory in bees [32–35]. Under controlled laboratory conditions, harnessed bees are trained to associate an odorant (the conditioned stimulus, CS) with a sucrose reward (the unconditioned stimulus, US). A bee that learns the association will extend its proboscis in response to the odor alone, in anticipation of a food reward. Honey bees learn such associations with remarkable efficiency and can remember them for weeks. These manifestations of behavioral plasticity are accompanied by changes in the activity patterns [36–40] and ultimately, the connectivity [41–42] of neural circuits in centers of the brain identified as key substrates for olfactory memory formation (for reviews: [31,43–45]).

In this study, we examine the functional properties of the honey bee allatostatin receptors A (Apime-ASTA-R) and C (Apime-ASTC-R), we identify where in the brain the genes that encode these receptors are expressed, and we investigate the effects of exogenously applied allatostatins A (Apime-ASTA), C (Apime-ASTC) and CC (Apime-ASTCC) on appetitive olfactory learning in the bee. Our results show that allatostatins modulate learning and memory in this insect, and provide important insights into the evolution of somatostatin/allatostatin signaling.

## Material and Methods

### Identification of *Apis mellifera* allatostatin-receptor genes

#### Sequence analysis

Protein sequence data from the improved honey bee genome assembly (Amel 4.5 [46]) were screened using BLAST (available at http://www.ncbi.nlm.nih.gov) for putative Apime-ASTA- and Apime-ASTC-like receptor sequences using sequences of known receptor genes for *Drosophila melanogaster* allatostatins A (known as DAR1, CG2872 and DAR2, CG10001) and C (Drostar1, CG7285, Drostar2 CG13702, CG14919). Two putative AST-receptor protein sequences were identified, GB43574 and GB55818 **(**formerly referred to as GB19021 and GB20155 respectively in the previous genome assembly, and predicted as allatostatin receptors [9]). We used the Splign Alignment Tool (available at http://www.ncbi.nlm.nih.gov) to detect introns in the genomic DNA sequences of these two putative AST-receptor genes, and BLAST to look for possible splice variants. We aligned the amino-acid sequences with Multiple Alignment using Fast Fourier Transform (MAFFT [47]), and used Phobius (http://www.phobius.sbc.su.se), tmap and toppred (both available at http://mobyle.pasteur.fr) to predict the trans-membrane domains. The consensus of the three methods was chosen and aligned to the published sequences of human opioid and somatostatin receptors (see for example [48]), as these receptors are the closest homologs to the *Drosophila* allatostatin receptors [49–50], and have been studied in great detail. We also used a prediction tool for intracellular coupling of the putative AST-receptor proteins to G proteins (http://bioinformatics.biol.uoa.gr/PRED-COUPLE), based on the algorithm used by [51]. To predict phosphorylation sites, we used NetPhosK Server [52].

#### Phylogenetic analysis

The alignment and the phylogenetic tree were done using either the Clustal Omega [53] or the MUSCLE software. Both methods gave the same phylogenetic tree, and the tree shown here was built using the Phylogeny.fr package [54]. After alignment, ambiguous regions (*i*. *e*. containing gaps and/or poorly aligned) were removed with Gblocks (v0.91b) using the following parameters: minimum length of a block after gap cleaning = 10, no gap positions were allowed in the final alignment, all segments with contiguous non-conserved positions bigger than 8 were rejected, minimum number of sequences for a flank position = 85%. The phylogenetic tree was reconstructed using the maximum likelihood method implemented in the PhyML program (v3.0). The default substitution model was selected assuming an estimated proportion of invariant sites and 4 gamma-distributed rate categories to account for rate heterogeneity across sites. The gamma shape parameter was estimated directly from the data. Reliability for internal branch was assessed using the bootstrapping method (199 bootstrap replicates) and the bootstrap value is indicated on each branch. Urotensin-2 receptor (UR2R) was chosen as an out-group for somatostatin and galanin receptors (SSTR and GALR) based on the phylogenetic tree described in [55]. Phylogenetic analysis with another out-group receptor, CXCR-5, which is more distant than UR2R, did not change the results. The accession number of the proteins are: NP_444474.1 (KISS1R), NP_032108.1 (GALR1), NP_034384.3 (GALR2), NP_033242.1 (SSTR1), NP_033243.2 (SSTR2), NP_033244.2 (SSTR3), NP_033245.2 (SSTR4), NP_035555.1 (SSTR5), NP_660114.1 (MCHR1), NP_034472.1 (NPBWR1), NP_663415.1 (UR2R); fruit fly: NP_524700 (ASTAR1), NP_524544 (ASTAR2), NP_649040.2 (Drostar1), NP_649039.4 (Drostar2).

### Characterization of the allatostatin receptors

#### Cloning of ASTA-R and ASTC-R

For heterologous expression of the putative Apime-ASTA- and Apime-ASTC-receptors, the coding region of each putative AST-receptor gene was inserted into the plasmid vector, pRK5. This vector contains an ATG initiation codon in phase with a mammalian signal peptide sequence of metabotropic glutamate receptor 5 to address receptor protein to the cell membrane, with a hemagglutinin sequence (TAG HA), and MluI and HindIII restriction sites used for cloning. The coding regions of the genes of interest were PCR amplified from cDNA template. After extraction from whole brains, 1 μg of total RNA was reverse-transcribed with random primers. PCR was performed with 2 μl of RT product, 1 μl of *Apime-ASTA-R-* or *Apime-ASTC-R*-specific forward and reverse primers (100 μM), 1 μl of dNTPs (10 mM, ThermoFischer) and 0.5 μl of Pfu DNA Polymerase (2,5 U/μl, ThermoFischer). PCR reactions included: 30s at 98°C, then 30 cycles of 10s at 98°C, 30s at 60°C, 1 min at 72°C and finally, 5 minutes at 72°C. Cloning required several amplification and digestion steps to insert the full cDNA sequences in the vector. The final PCR products were digested with MluI and HindIII and the resulting fragments were cloned into the plasmid vector. pRK5-*Apime-ASTC-R* and pRK5-*Apime-ASTA-R* were purified with MaxiPrep kit (Qiagen) and sequenced.

#### Cell transfection

For functional assays, human embryonic kidney (HEK293) cells were maintained in Dulbecco's Modified Eagle’s Medium nutrient mixture F12-Ham (DMEM/F12, Sigma–Aldrich) supplemented with 10% heat-inactivated fetal bovine serum (Gibco), 100 IU/ml penicillin, 100 μg/ml streptomycin (Gibco).

For intracellular calcium measurements, exponentially growing cells (ca. 10^7^ cells per transfection) were transfected with 10 μg of plasmid construct DNA (pRK5-*Apime-ASTC-R* or pRK5-*Apime-ASTA-R*) encoding *Apime-ASTA-R* (5 μg) or *Apime-ASTC-R* (5 μg) using electroporation. In all cases, 2 μg of plasmid DNA encoding the Gα_qi9_ chimeric G protein was included to enable artificial coupling of the receptors to phospholipase C. Electroporation (250 V, 800 μF, Biorad GenePulser X-cell) was performed in a total volume of 300 μl of electroporation buffer (50 mM K_2_HPO_4,_ 20 mM CH_3_COOK, 20 mM KOH, 26.5 mM MgSO_4_, pH 7.4). After electroporation, cells were resuspended in 10 ml of culture medium and split (100 μl/well) into polyornithine-coated (Sigma #P3655), black-walled, clear bottom 96-well plates (Greiner bio-one #655090) and grown for a further 48 h prior to analyzing receptor properties. All products used for cell culture were purchased from Invitrogen Life Technologies.

For intracellular cAMP measurements, cells were bathed in trypsin EDTA and collected, then suspended in fresh DMEM. Transfections with pRK5-*ApimeAstC-R*, pRK5-*ApimeAstA-R* or empty pRK5 vector were carried out in 15 ml Falcon tubes. Transfection medium for HEK293 cells was prepared using the Lipofectamine LTX kit (Invitrogen) with 2.5 ml Opti-MEM® (Gibco), 12.5 μl Plus™ Reagent and 4 μg of ApimeASTC-R (or ApimeASTA-R, or empty) expression construct, and 2 μg of CRE(6x)-Luc plasmid [58]. The luciferase ORF, downstream of six tandem repeats of a cyclic AMP responsive element (CRE), in front of a minimum collate promoter, was used as a reporter gene (this reporter plasmid has been used by others [56–58])

#### Intracellular Calcium Measurements

Cells were washed with freshly prepared 20 mM Hepes, 1 mM MgSO_4,_ 3.3 mM Na_2_CO_3,_ 1.3 mM CaCl_2_, 0.1% BSA, 2.5 mM probenecid in 1X Hank's balanced salt solution (Gibco #14185), pH7.4 buffer and loaded with 1 μM Ca^2+^-sensitive fluorescent dye Fluo-4 AM (Invitrogen # F14202) for 1 h at 37°C. Cells were washed once and incubated with 50 μl of buffer. A plate was prepared with the various concentrations of peptides to be tested, and 50 μl of 2X concentrated-drug solution was added in each well after 20 s of recording. Fluorescence signals (excitation 485 nm, emission 525 nm) were measured by using the fluorescence microplate reader Flexstation (Molecular Devices) at sampling intervals of 1.5 s for 60 s. All represented data correspond to means ± standard deviation (SD) from experiments performed in triplicate.

#### Intracellular cyclic AMP (cAMP) reporter assay

We followed a previousy published protocol [58]: HEK293 cells in Dulbecco's Modified Eagles Medium nutrient mixture F12-Ham (DMEM/F12, Sigma–Aldrich) supplemented with 10% heat-inactivated fetal bovine serum (Gibco), 100 IU/ml penicillin, 100 μg/ml streptomycin (Gibco) were maintained in an incubator at 37°C with constant supply of 5% CO_2_, until they reached 60–80% confluence. Cells were then bathed in trypsin EDTA (Gibco) until rounded. The flask was then tapped to loosen the cells, which were subsequently washed off with 5 ml DMEM/F12. The cells were gently centrifuged at 800 rpm for 5 minutes and carefully suspended in fresh DMEM, which was divided in two for transfection with receptor constructs or empty vectors. After 5 min at room temperature, 30 μl LTX was added. After an incubation period of 30 min at room temperature, the DNA/LTX mix was added to the cells. The cells were then aliquoted (100 μl per well) into a clear bottom 96-well plate (Greiner bio-one #655090) and incubated overnight (37°C, 5% CO_2_).

Prior to measuring cAMP, the cell medium was removed by carefully inverting the 96-well plate on filter paper and quickly replaced with 100 μl of DMEM/F12 without phenol red (Invitrogen), but containing 200 μM 3-isobutyl-1-methylxanthine (IBMX, Sigma–Aldrich) to prevent cAMP breakdown, as well as drugs to be tested. In each experiment, some wells were used to measure basal levels of cAMP in the cells (negative control), while others were used to measure cAMP levels in cells exposed to 10 μM forskolin analogue (NKH-477, Sigma Aldrich, positive control). In the remaining wells, Apime-ASTA, Apime-ASTC or Apime-ASTCC at a concentration ranging between 10^-3^M and 10^-14^M was added to the medium bathing the cells. All cells were incubated at 37°C and 5% CO_2_ for 3–4 h to allow transcription of the reporter gene before 100 μl of freshly reconstituted Steadylite Plus™ substrate (PerkinElmer) was added to each well. The 96-well plate was then placed in the dark for 15 min before light emission resulting from luciferase enzymatic activity was recorded. Measurements lasting 0.2 s/well were taken every 55 s for 10 cycles using a FLUOstar Omega microplate reader (BMG Labtech). Results were analyzed by means of the Omega software packages and further processed by Excel and GraphPad Prism softwares. In order to facilitate comparisons across experiments, bioluminescence levels are expressed relative to those in mock-transfected cells (transfected with the vector devoid of the receptor sequence) untreated with NKH-477, which were arbitrarily set at 100.

### Allatostatin signaling *in vivo*

#### Animals

In the morning of each day of experiments, honey bees *(Apis mellifera)* were collected as they were leaving hives or when they were returning to the hives with pollen loads (pollen foragers) either in research apiaries in Toulouse (France) or in Dunedin (New Zealand). Thus, most (if not all) individuals analyzed in this study were foragers. Our hives are used solely for research purposes so no specific permission was required to sample animals. The bees were then anesthetized briefly on ice before manipulation.

#### Apime-ASTA, Apime-ASTC and Apime-ASTCC gene expression in the brain

Brains were dissected in order to separate the optic lobes, as well as the ventral and dorsal halves of the central brain (containing respectively the antennal lobes and the mushroom bodies). Brain samples were immersed in liquid nitrogen and kept at -80°C until RNA extraction, which was performed using a Qiagen RNeasy Mini kit. DNAse treatment was applied using Turbo DNAse (Ambion 2238G). RNA was assessed for quantity and quality using a Nanodrop (Thermo scientific). Reverse transcription (RT) was performed using a RevertAid H Minus First Strand cDNA synthesis kit using both random hexamers and oligo dT (Thermo scientific) on 1 μg of total brain RNA. Polymerase chain reaction (PCR) was carried out using the primer pairs listed in S1 Table and Taq Polymerase (MP Biomedicals), with an annealing temperature of 52°C. The PCR products were analyzed via 1.5% agarose gel electrophoresis. A single band of the expected size was observed for each transcript, the identity of which was confirmed by sequencing.

#### Quantification of the allatostatin genes in the dorsal brain region

Brains were collected from pollen foragers, and their dorsal regions (including calyces and peduncle) dissected quickly, frozen on dry ice and stored at –80°C until RNA extraction, then homogenized individually in Trizol. RNA was isolated using RNA easy Mini purification kit (Qiagen) and RNA concentrations were measured using Quant-iT Ribo Green RNA assay (Invitrogen).

Real-time quantitative PCR (qPCR) analysis was performed as previously described [59]. 50 ng of RNA was reverse-transcribed using VILO Supercript (Invitrogen). Gene-specific amplification products were generated using ExpressSYBR® GreenER qPCR SuperMix (Invitrogen) and gene specific primer pairs (S2 Table). Assay efficiencies were derived from standard curves generated using pooled cDNA from all the bees. We used the ΔΔCt method with assay efficiencies incorporated in the formula to calculate transcript abundances: Normalized = (1 + Efficiency target, ^- ΔCt target^) / (1 + Efficiency reference, ^- ΔCt reference^). Transcript levels were normalized using the geometric mean of two reference genes, Am18S and elongation factor-1 alpha elf1α, as they show the smallest variation of all potential housekeepers tested (NormFinder).

#### Distribution of *Apime-ASTA-R* and *Apime-ASTC-R* expressing cells in the brain

*In situ* hybridization was used to examine the distribution of cells expressing *Apime-AstA-R* and *Apime-AstC-R* mRNAs in the adult honey bee brain. Pollen foragers were collected and cold anaesthetized before cutting a window in the head capsule of each bee to expose their brain. The brains were pre-fixed by adding a drop of 4% buffered paraformaldehyde (PFA) into the head capsule. The brains were then removed from the head capsule and fixed in 4% PFA at room temperature for 2 h, and then at 4°C overnight. They were then dehydrated and embedded in paraffin.

Freshly cut sections of brain tissue (5 μm thick) were mounted on polysine slides (BDH Laboratory Supplies). The sections were treated with xylene and rehydrated before proceeding with *in situ* hybridization following a modified version of the protocol from [60], as described by [61]. Digoxygenin (DIG)-labeled sense and antisense ribo-probes were synthesized according to [62] and used at a concentration of 2 mg/ml. Anti-DIG antibody (1:5000, Roche Molecular Biochemicals) was applied to the brain sections for 2 hours. The sections were then stained with 4-nitro blue tetrazolium chloride (450 mg/ml) and X-phosphate/5-bromo-4-chloro-3-indolyl-phosphate (175 mg/ml) (Roche Molecular Biochemicals) for 16–18 h at room temperature. Staining was stopped with two 10-min washes in distilled water. Nonspecific staining was removed by washing in 95% ethanol for up to 30 min. Sections were rehydrated in distilled water and mounted using Aquamount (BDH Laboratory Supplies).

#### Receptor binding assays

Apime-ASTA radiolabel was generated through radio-iodination in the presence of chloramin T. Mono-iodinated derivative was purified by reverse phase HPLC on a C-18 column to a specific activity of 2000 Ci/mmol. Binding assays were performed using ^125^I-allatostatin A (100–150 pM) according to the protocol previously described in detail [63]. The binding buffer was composed of DMEM with 0.5% bovine serum albumin (BSA).

For binding experiments on crude bee brain membranes, an aliquot of membranes (35–55 μg proteins) was incubated 1 hour at room temperature with ^125^I-Apime-ASTA (100–150 pM) alone or in the presence of competitor in Tris 50 mM pH = 7, 1 mM ethylene glycol tetra-acetic acid, 5 mM MgCl_2_, 0.05% soybean trypsin inhibitor, 0.1 mM phenyl methane sulfonyl fluoride (PMSF), 0.25 mg/ml bacitracin, 0.1% BSA. Then, binding was stopped by centrifugation 5 min at 12,000 RPM at 4°C. Pellets were washed with 1 ml of cold Tris 50 mM with 1% BSA buffer before radioactivity determination. IC_50_, which corresponded to the concentrations of competitor inhibiting 50% of specific binding, were calculated using the non-linear curve fitting software GraphPad Prism.

#### Effects of allatostatins on appetitive olfactory learning performance

For behavioral experiments, bees departing from the hive (mostly foragers) were chosen as they usually show good motivation and learning ability. Each cold-anesthetized animal was introduced into a metal holder and fixed to it in order to restrain all movements of the appendages apart from those of the antennae and proboscis. Each bee was then fed with 5 μl of a sucrose solution (50% w/w in water), and its median ocellus was removed to allow injection of allatostatin into the head capsule. All bees were then placed in a dark, warm (25°C) and humidified compartment for 3 hours, before the experiments began.

#### Allatostatin treatment

The allatostatin peptides **(dry powder, >95% pure)**, Apime-ASTA, Apime-ASTC and Apime-ASTCC were synthesized by GeneCust (Luxemburg). In the case of Apime-ASTA, for which several isoforms exist, we chose to use the one reported to be the most abundant in the bee brain (GRQPYSFGL-amide: [13–14]). Apime-ASTC (SYWKQCAFNAVSCF-amide) and Apime-ASTCC (GQAKGRVYWRCYFNAVTCF) were both cyclized between their cysteine residues. The vehicle for allatostatins was a saline solution made of PBS containing the protease blocker, 1 mM PMSF (prepared from a 30mM stock solution in 100% ethanol, final concentration of ethanol 3.3%). PMSF was found in preliminary HPLC experiments to prevent allatostatin degradation in the haemolymph (data not shown). Bees injected with vehicle only (PBS plus 1 mM PMSF) were used as controls (blocking protease activity does not affect bees’ learning ability [64]).

The allatostatins were injected into the head haemolymph after penetrating the median ocellus, either 1 hour before olfactory conditioning or sucrose responsiveness assessment, or immediately after olfactory conditioning. All injections were performed under a binocular microscope. A micromanipulator-controlled Hamilton syringe (Nanofil, equipped with a NF33BV2 needle, WPI) was used to inject approximately 200 nl of peptide solution. To make sure that the solution was properly injected, it was pushed out of the syringe so that a drop was visible and then the drop was put in contact with the haemolymph where it was quickly absorbed thanks to the respiratory movements of the bee. Any bee with excessive bleeding was discarded.

#### Olfactory conditioning

Associative conditioning of the proboscis extension reflex was used to monitor effects of allatostatin treatment on appetitive olfactory learning performance. Olfactory stimuli were prepared shortly before conditioning by inserting a small piece of filter paper soaked with 4μl of pure odorant (from Sigma-Aldrich, Lyon, France) in one of the 8 channels of a custom-made, computer-driven olfactometer (P. Arrufat, Research Center on Animal Cognition, Toulouse, France). Immediately before conditioning, the integrity of the proboscis extension reflex was checked by touching both antennae with a 50% (w/w in water) sucrose solution. Olfactory conditioning of this reflexive response was carried out following a standard protocol [35] consisting of three paired presentations of the odor (conditioned stimulus, CS) with 50% sucrose (the unconditioned stimulus, US) separated by a 10-min interval. Each trial included a familiarization phase in which the bee was placed in the conditioning set up for 13 s before forward pairing of the CS-US. The CS (1-nonanol) was delivered to the bee through an airflow for 4 s, and the US (sucrose solution) for 3 s, with a 1 s CS-US overlap. The US was presented by gently touching the antennae to elicit the PER; as soon as the bee extended its proboscis it was allowed to lick the toothpick. This sequence was controlled by the computer-driven olfactometer. Conditioned responses (CR) were defined as extensions of the proboscis further than the mandibular tips that occurred when the odor (CS) was presented, but before the sucrose reward (US) was delivered; bees that showed CR were rewarded on the proboscis directly. Any bee that failed to display reflexive proboscis extension in response to sucrose stimulation of the antennae before or during conditioning was discarded.

#### Sucrose responsiveness assessment

Bees were injected with saline, Apime-ASTA, Apime-ASTC or Apime-ASTCC (1μM, as described above), 1h before the test. Since sucrose was presented diluted in water, bees were given water *ad libitum* immediately before testing to ensure that their responses would be elicited by sucrose only. We followed a published protocol [65]: six sucrose solutions of increasing concentration (0.1%, 0.3%, 1%, 3%, 10% and 30%) were presented in succession to the antennae, interspersed with water stimulations to avoid sensitization. For each animal, the presence or absence of a proboscis extension response to each stimulus was recorded. A vast majority of bees had consistent responses, *i*.*e*. when they responded to a given concentration, they also responded to higher concentrations. Those bees not responding to the highest sucrose concentration tested for (30%) had their reflexive response to sucrose checked using a 50% sucrose solution as a positive control (as for conditioning). Any bee that failed to display proboscis extension at this concentration was discarded.

#### Olfactory generalization test

Bees were conditioned as previously described and were immediately injected with saline or one allatostatin (1μM Apime-ASTA, Apime-ASTC or Apime-ASTCC). Bees that learned to respond to the conditioned stimulus (CS) at the end of conditioning were submitted to a generalization test 1h later. In addition to 1-nonanol (the CS), two novel odorants (nonanal and 1-hexanol) were presented: these were selected according to their respective high and low levels of perceptual similarity with 1-nonanol [66]. All odorants were presented without reinforcement with sucrose. The order of odorant presentations varied across individuals, and the integrity of the reflexive response to sucrose was checked at the end of the test. Bees that failed to display the reflex were discarded.

### Statistics

Functional data (receptor-mediated calcium, cAMP signals and binding) were plotted and regression curves were fitted using the GraphPad Prism software (San Diego, CA). The learning data were analyzed with generalized linear effects modeling (GLM) for binomial data using the R package lme4 [67]. Response levels recorded in the last conditioning trial were used to assess the effect of treatment on learning. For 1-hour memory and generalization tests, effects of allatostatin treatment were compared for each odor tested. Generalized linear mixed effects modeling (GLMM) was also used to analyze responsiveness to sucrose, with concentration and treatment as fixed factors and the bee identity and solution presented (either sucrose or water) as random factors. Results are presented as coefficients ± standard error. All statistics were generated in R [68].

## Results

### Putative Apime-ASTA and Apime-ASTC receptors are close relatives of galanin and somatostatin receptors, respectively

**Fig 1:**
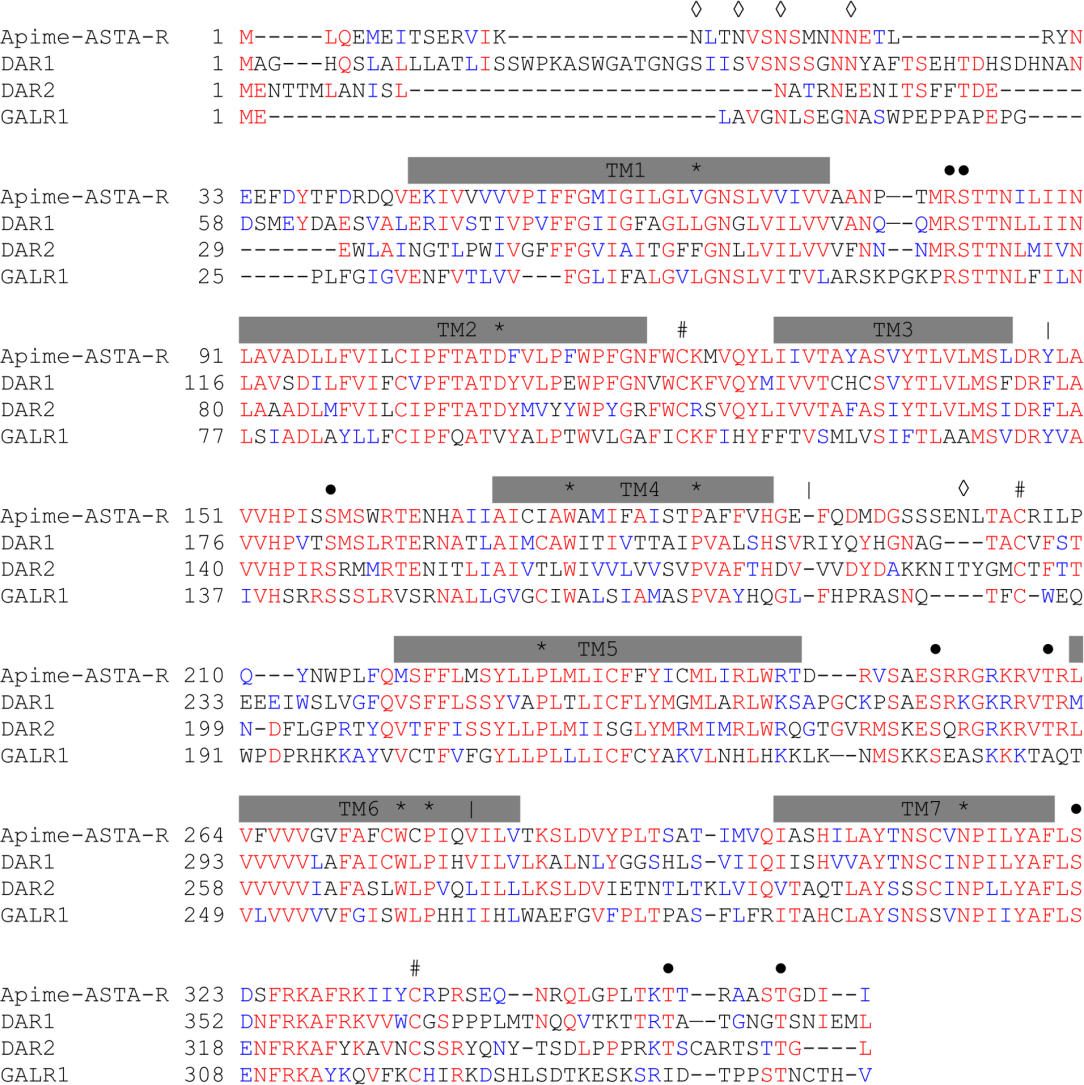
Aminoacid sequence alignment of Apime-ASTA-R and its *Drosophila* and human homologs. Sequence alignments of the predicted allatostatin A receptor in the honey bee *Apis mellifera* (Apime-ASTA-R, GenBank: XP_006560262.1) and of its homologs in the fruit fly *Drosophila melanogaster* (DAR1, GenBank: NP_726877.2 and *DAR2*, GenBank: NP_524544.1) and human (GALR1, NP_001471.2). The amino acid position is indicated on the left. Conserved residues are in red, and conservative changes in blue. Vertical bars (|) indicate locations of putative introns; grey bars indicate putative trans-membrane regions (TM1–TM7). Amino acids that are characteristic of class A GPCRs are indicated by *, open diamonds (◊) indicate putative N-linked glycolysation sites, # indicate cysteine residues for disulfide bridge (between TM2—TM3 and TM4 –TM5) or palmitoylation (intracellular domain), and dots (●) indicate conserved putative phosphorylation sites for PKA/C. Sequences are based on transcripts (cDNA).

**Fig 2:**
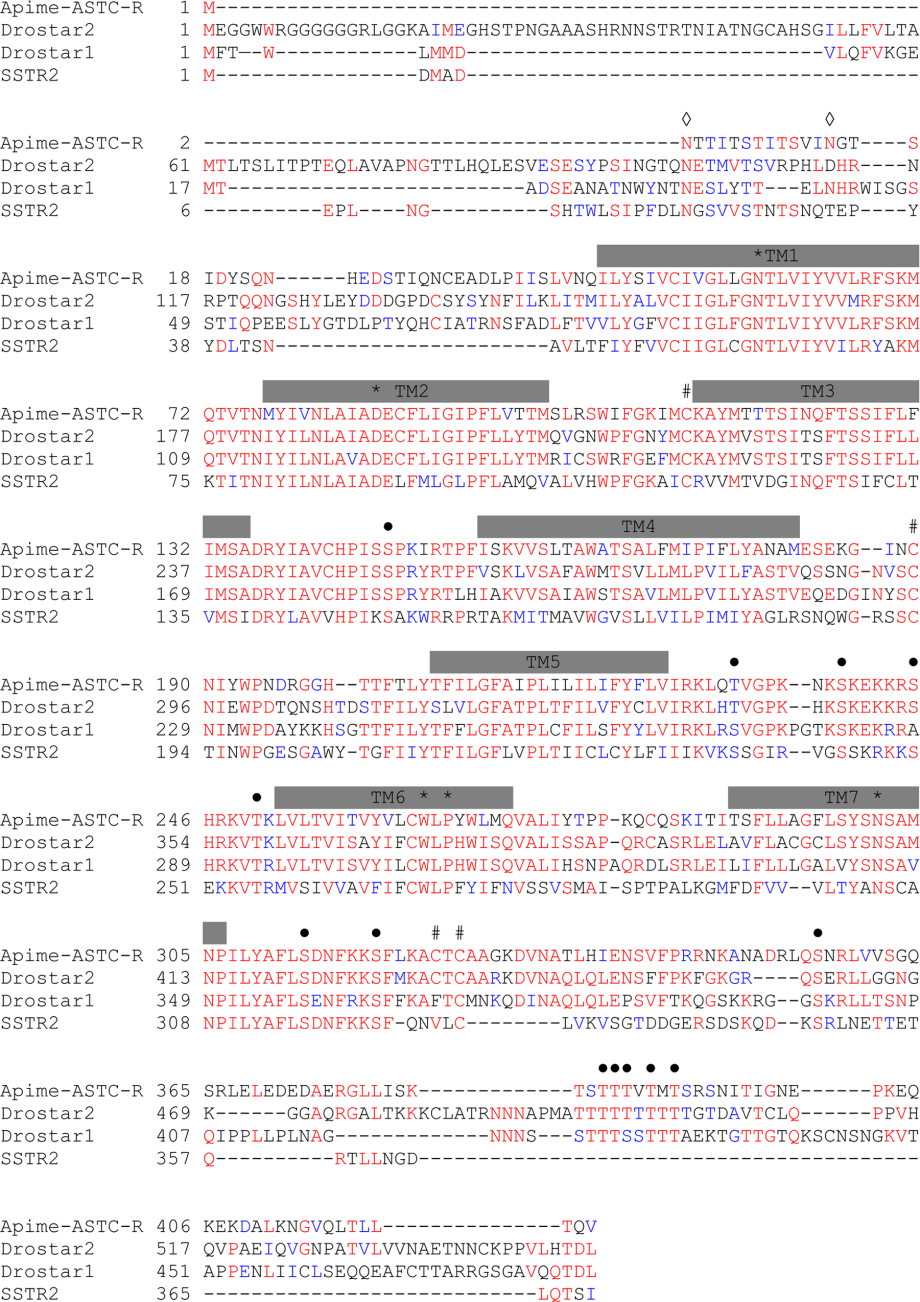
Aminoacid sequence alignment of Apime-ASTC-R and its *Drosophila* and human homologs. Sequence alignments of the predicted allatostatin C receptor in the honey bee *Apis mellifera* (*Apime-ASTC-R*, GenBank: XP_006560939.1) and of its homologs in the fruitfly *Drosophila melanogaster* (*Drostar1*, GenBank: NP_649040.2 and *Drostar2*, GenBank: NP_649039.4), and human (SSTR2, NP_001041.1). The amino acid position is indicated on the left. Conserved residues are in red, and conservative changes in blue. Grey bars indicate putative trans-membrane regions (TM1–TM7). Amino acids that are characteristic of class A GPCRs are indicated by *, open diamonds (◊) indicate putative N-linked glycolysation sites, # indicate cysteine residues for disulfide bridge (between TM2—TM3 and TM4 –TM5) or palmitoylation (intracellular domain), and dots (●) indicate conserved putative phosphorylation sites for PKA/C. Sequences are based on transcripts (cDNA).

To identify putative ASTA- and ASTC-receptors in the honey bee, we screened the honey bee genome with sequences of known receptor genes for *Drosophila melanogaster* allatostatins A (DAR1, CG2872 and DAR2, CG10001) and C (Drostar1, CG7285, Drostar2 CG13702, CG14919). Based on earlier studies, it was predicted that there would be no allatostatin CC-specific receptor [19]. Though *Drosophila* has two A-type and two C-type receptor genes, we identified only two sequences in the honey bee genome that provided matches, one for each receptor type. The first sequence (GB43574, known as GB19021 in the previous genome assembly) shares 51% and 45% amino-acid identity with *DAR1* and *DAR2*, respectively, and thus is referred to here as a putative Apime-ASTA-receptor gene (*Apime-AstA-R*, Fig 1). Three mRNA transcript variants exist for *Apime*-*AstA-R* as the genomic region contains several introns (Fig 1), but all result in the same cDNA (1083nt) and protein sequence (361AA, calculated mass: 41.23 kDa). The second sequence (GB55818, known as GB20155 in the previous genome assembly) shares 53% and 51% amino acid identity with *Drostar 1* and *Drostar 2*, respectively, the genes that encode *Drosophila* ASTC receptors, and will be referred to as *Apime-AstC-R*. Hence the protein encoded for by this second gene is referred to here as Apime-ASTC-R (Fig 2). The corresponding genomic sequence contains no introns (1272 bp) and the resulting protein is predicted to contain 421 amino-acids (calculated mass: 47.47 kDa).

**Fig 3:**
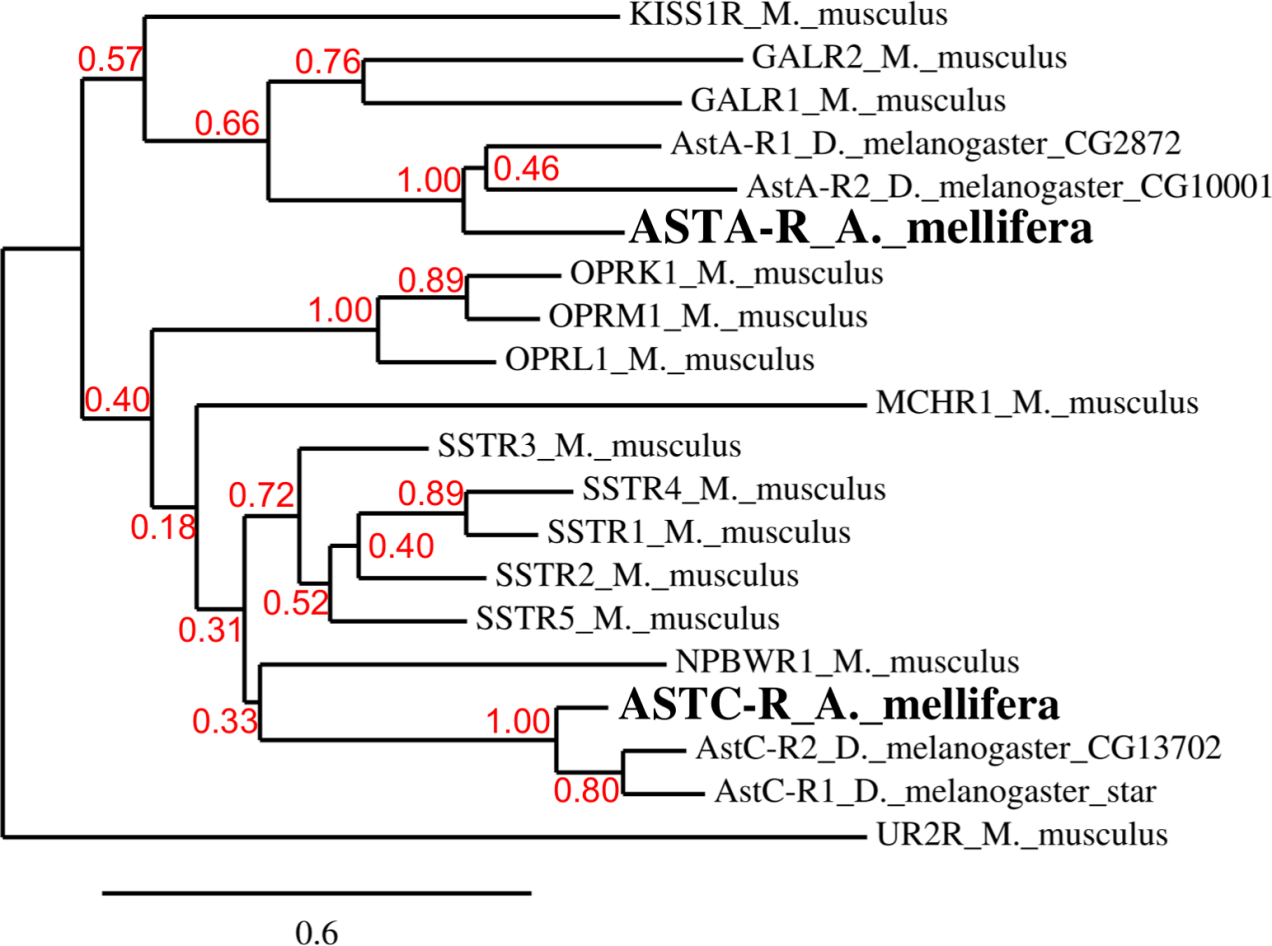
Phylogenetic tree of *A*. *mellifera* and *D*. *melanogaster* allatostatin receptors with respect to their closest mouse homologs. *Drosophila* gene names are indicated. For *M*. *musculus* receptors, abbreviations are: GALR (Galanin receptor), KISS1R (kisspeptin receptor), OPR (opioid receptor), MCHR (melanin concentrating hormone receptor), NPBWR (Neuropeptide B and W receptor), SSTR (somatostatin receptor). The bottom bar indicates the phylogenetic distance scale. The bootstrap values are displayed in red.

Phylogenetic analysis confirmed that the identified honey bee sequences share homologies with a variety of class A mammalian GPCRs. More specifically, this analysis (Fig 3) clearly shows that the putative honey bee ASTA- and ASTC-receptors, like their *Drosophila* counterparts, have galanin and somatostatin receptors, respectively, as their closest relatives. The closest mouse homolog for Apime-ASTA-R is the galanin receptor GALR1 (36.3% identity, 72.3% similarity) and that of Apime-ASTC-R is the somatostatin receptor SSTR2 (34.9% identity, 63.9% similarity). Thus, A- and C-type allatostatins of *A*. *mellifera*, like those of *D*. *melanogaster*, appear to belong to different, yet related families.

Both honey bee amino-acid sequences of interest (GB43574 and GB558818) are predicted to display the characteristic seven trans-membrane (TM) regions of GPCRs (Figs 1 and 2). They also contain consensus motifs for potential N-glycosylation sites in the extracellular N-terminus [69] and for phosphorylation by protein kinases A and C in the intracellular loops [52,70]. Like their fruit fly counterparts, they share most characteristics of class A GPCRs [71], including the DRY motif on the second intracellular loop, which is key for receptor activation [72], as well as several characteristic amino-acids that are important for the conformation of the receptors [71] (Figs 1 and 2), notably an asparagine in TM7, which, along with the aspartic acid in TM2, permits a hydrogen bond between these two TM domains. Also, two cysteines belonging to extracellular loops 2 and 3, likely form a disulfide bond important for receptor structure and ligand binding [73]. Finally, the C-terminal end contains a conserved cysteine to which a palmitic acid can be attached and used as an anchor on the inner surface of the plasma membrane [74]. Altogether, these properties point to two functional GPCRs in honey bees, one for ASTA and another for ASTC and CC. Using an algorithm developed by [51], we predicted that Apime-ASTA-R and Apime-ASTC-R would couple to G*α*_*i/o*_ and inhibit the production of cAMP from ATP. To test this prediction, we expressed the two receptors separately in HEK293 cells together with either a chimeric G protein G*α*_*qi9*_ or with the luciferase reporter gene.

### Apime-ASTA-R and Apime-ASTC-R are functional and exhibit ligand specificity

**Fig 4:**
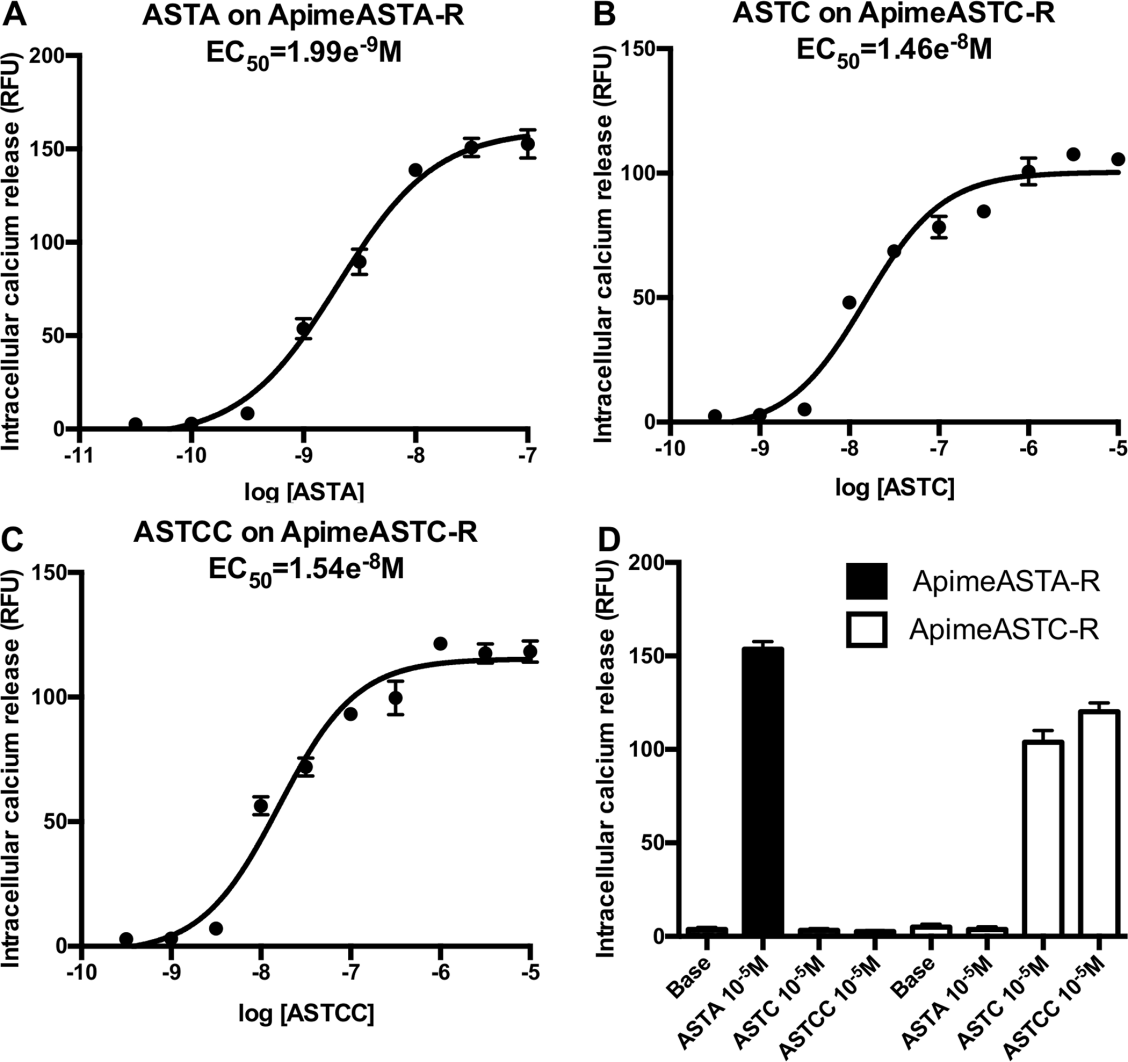
Functional characterization of Apime-ASTA and Apime-ASTC receptors *in vitro*. HEK293 cells were transiently transfected with Apime-ASTA or Apime-ASTC receptors and a chimeric G protein allowing artificial coupling to PLC (see materials and methods). Receptor activation following stimulation by different concentrations of peptides was determined by monitoring intracellular calcium levels (indicated by relative fluorescence units, RFU). Concentration-response curves of Apime-ASTA on Apime-ASTA-R (A), Apime-ASTC on Apime-ASTC-R (B), and Apime-ASTCC on Apime-ASTC-R (C). Numbers below the X axis indicate the molar concentration (M). (D) Apime-ASTA is a specific ligand of Apime-ASTA-R; Apime-ASTC and Apime-ASTCC are specific to Apime-ASTC-R (means +/- SD from one representative experiment performed in triplicate). Base: calcium levels without peptide. Curves were obtained by fitting the data using nonlinear regression analysis in the GraphPad Prism software.

In order to confirm that the identified sequences correspond to functional receptors for allatostatins, we expressed Apime-ASTA-R and Apime-ASTC-R separately in HEK293 cells together with G*α*_*qi9*_ chimeric G protein, thus allowing artificial coupling of the receptors to phospholipase C. This method allows detecting ligand-induced activation of the receptor by measuring the release of intracellular calcium. Applying increasing doses of the appropriate allatostatin to cells expressing Apime-ASTA-R or Apime-ASTC-R induced dose-dependent increases in calcium release that were absent in cells not expressing these receptors (Fig 4). More specifically, ASTA was able to activate Apime-ASTA-R with an EC_50_ of 1.99 nM (Fig 4A), but this receptor was not activated by either ASTC or ASTCC (Fig 4D). In contrast, both ASTC and ASTCC activated Apime-ASTC-R with EC_50_ values of 14.6 nM and 15.4 nM, respectively (Fig 4B and 4C), but neither peptide activated Apime-ASTA-R (Fig 4D). Thus, both receptors *in vitro* are activated by specific allatostatins, and no cross-activation was detected. No calcium release was detected when SOM230 (a potent somatostatin receptor agonist, at concentrations up to 0.1mM) was applied to Apime-ASTA-R or Apime-AST-CR transfected cells (data not shown).

### Receptor expression lowers levels of intracellular cAMP

**Fig 5:**
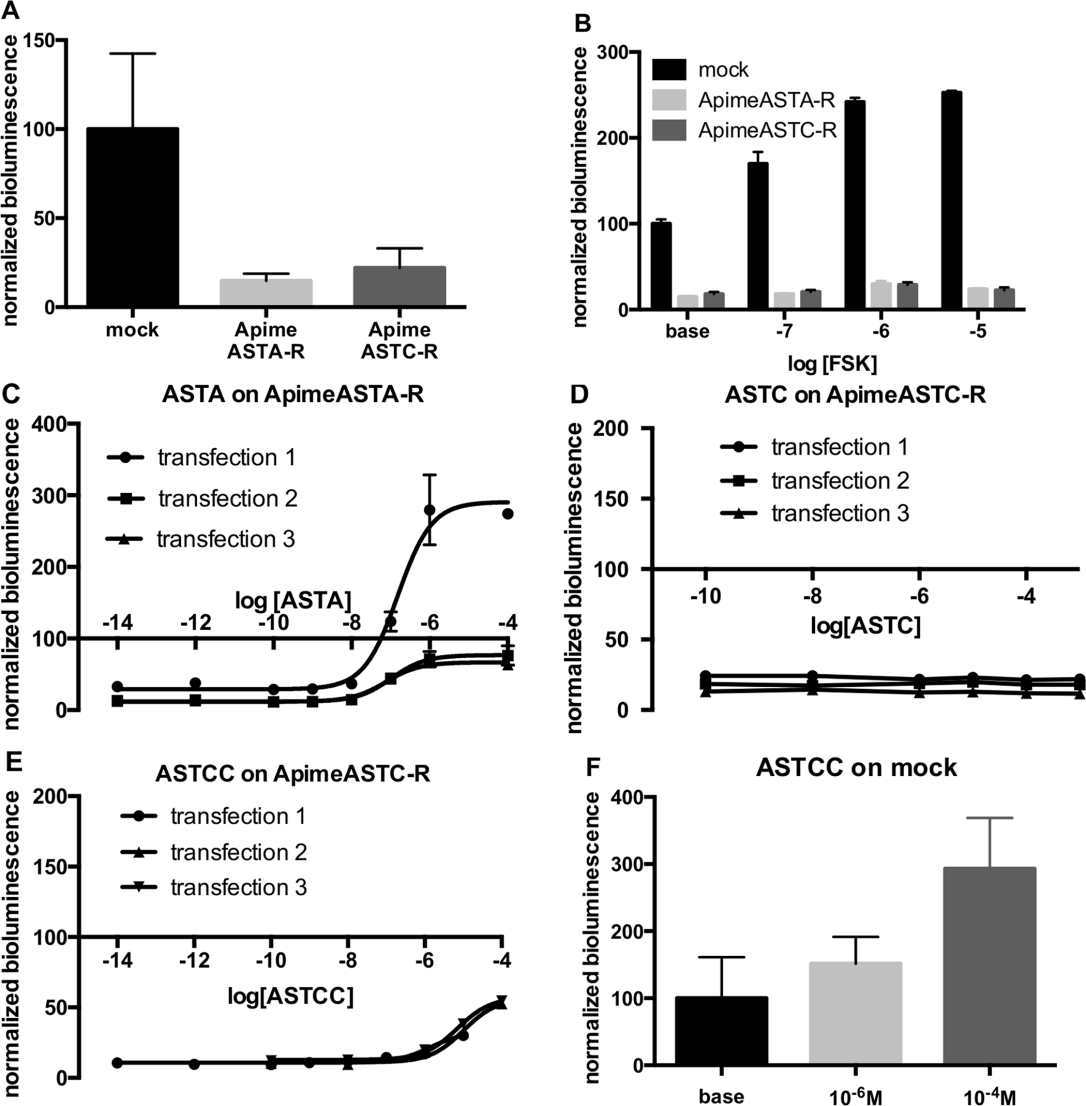
Apime-ASTA-R and Apime-ASTC-R display constitutive inhibitory activity, down-regulating cAMP. HEK cells transfected with Apime-ASTA-R or Apime-ASTC-R show lower levels of bioluminescence than mock-transfected cells (A). Bars represents mean+/-SD values of 8 transfections in duplicates (mock) or triplicates (Apime-AST-R). Forskolin analog NKH-477 has a reduced ability to increase bioluminescence levels in Apime-ASTA-R and Apime-ASTC-R transfected cells than in mock-transfected cells (B). Bars represent mean +/- SD values of 2 transfections in duplicates or triplicates. Application of increasing doses of Apime-ASTA on Apime-ASTA-R transfected cells induced a dose-dependent increase in bioluminescence (C). Application of increasing doses of Apime-ASTC on Apime-ASTC-R transfected cells did not change bioluminescence levels (D). Application of increasing doses of Apime-ASTCC on Apime-ASTC-R transfected cells increased bioluminescence levels at high concentrations (E). HEK cells transfected with luciferase reporter gene and an empty vector (mock-transfected) show a dose-dependent response to Apime-ASTCC (F). Bars represent mean +/- SD values of 3 transfections in triplicates.

The activation of G*α*_*qi9*_ protein shown in the previous experiment suggests that both allatostatin receptors are naturally coupled to the G*α*_*i*_ subtype, which decrease cAMP production via adenylyl cyclase inhibition. To test this prediction, we expressed the two receptors separately in HEK293 cells together with the luciferase reporter gene, downstream of the cAMP response element, CRE, but without exogenous G proteins. We found that estimated levels of cAMP in cells expressing either Apime-ASTA-R or Apime-ASTC-R were 85.2% and 78% lower respectively than in cells transfected with an empty vector (Fig 5A, see also 5B–5E), suggesting that even in the absence of allatostatin, adenylyl cyclase is inhibited by the presence of the allatostatin-receptor proteins.

To examine this inhibition further, we exposed cells to forskolin analog, which generally activates adenylyl cyclase and increases intracellular levels of cAMP (Fig 5B). In cells transfected with an empty vector, the forskolin analog NKH-477 (10 nM to 10 μM) increased bioluminescence in a dose-dependent manner (2.5-fold increase with 10 μM compared to baseline), as expected. At the same concentrations, however, forskolin analog had a smaller effect on cAMP levels in cells expressing Apime-ASTA-R (1.59-fold increase), or Apime-ASTC-R (1.24-fold increase).

We then applied increasing concentrations of either type of Apime-ASTs on Apime-ASTA-R and Apime-ASTC-R transfected cells, respectively (Fig 5C–5E). For purposes of comparison, bioluminescence levels in the cells were normalized to the levels recorded in mock-transfected cells. Opposite to what was observed in Apime-ASTA-R-transfected cells with respect to mock cells (presumably due to constitutive activity), adding Apime-ASTA to these same transfected cells induced a dose-dependent increase in cAMP levels, with a mean EC_50_ = 103.4 nM (Fig 5C). In one of 3 independent transfections, cAMP levels in cells treated with concentrations of Apime-ASTA greater than 100 nM exceeded the levels detected in mock-transfected cells, but in the other two transfections, Apime-ASTA-induced increases in bioluminescence peaked at levels close to those detected in mock-transfected cells.

Applying Apime-ASTC to Apime-ASTC-R-transfected cells (Fig 5D) did not change the bioluminescence levels, whereas at high concentrations (>10 μM), Apime-ASTCC applied to Apime-ASTC-R-transfected cells induced a small increase in intracellular cAMP (Fig 5E). However, a similar response to this peptide was apparent also in mock-transfected cells (Fig 5F), suggesting that this effect was not dependent on the expression of Apime-ASTC-R. No effect of Apime-ASTC and Apime-ASTA were detected in mock-transfected cells (data not shown).

### Apime-ASTA-R and Apime-ASTC-R are present in the honey bee brain

**Fig 6:**
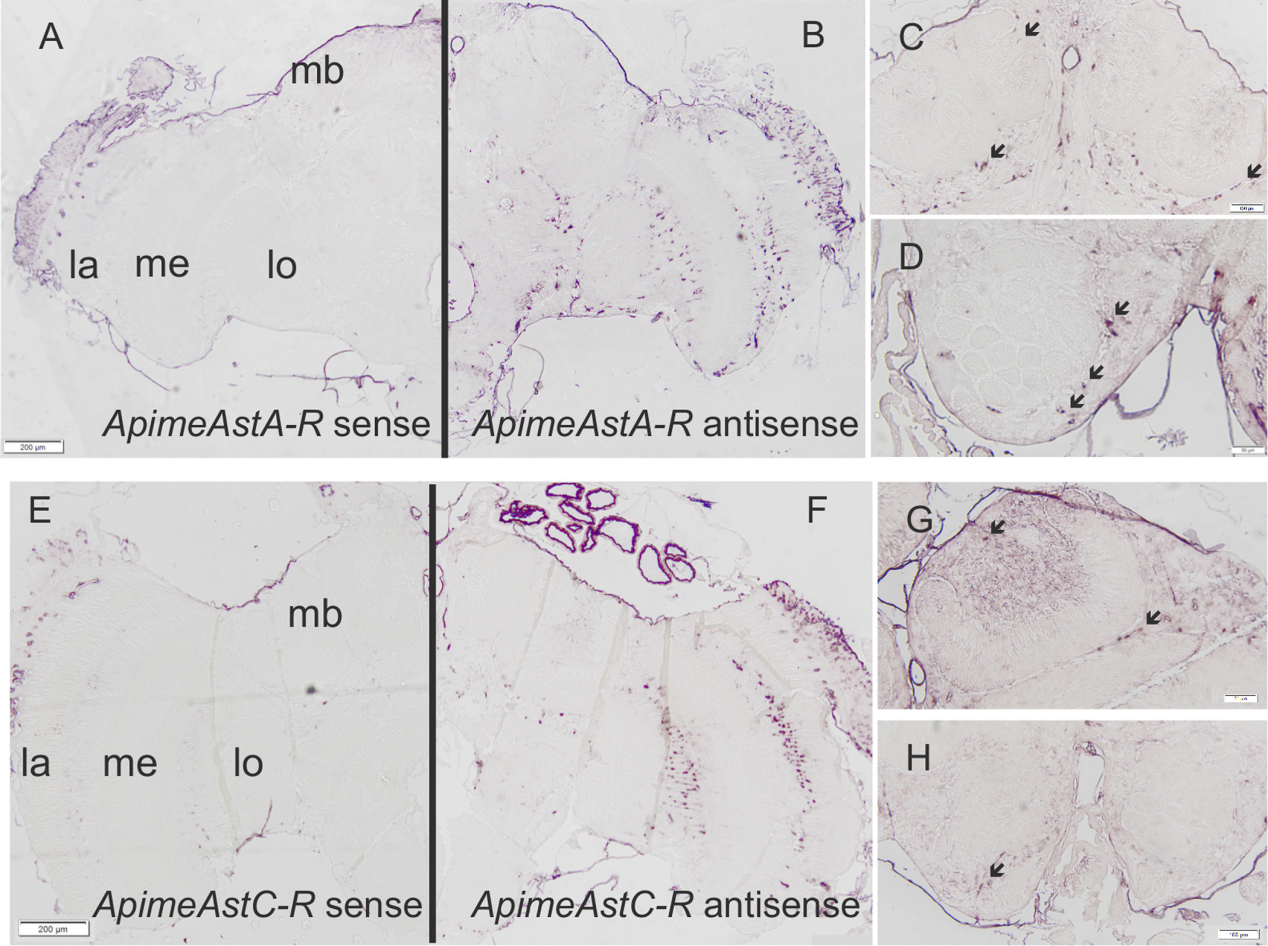
*Apime-AstA-R* and *Apime-AstC-R* mRNAs are expressed in the brain. *In situ* hybridizations were performed with antisense probes specific for *Apime-AstA-R* (B, C, D) or *Apime-AstC-R* (F, G, H) on sections from pollen forager brains. Sense probes revealed no specific staining (A, E). Specific hybridization was detected in the optic lobe, and particularly between the lamina (la), medulla (me) and lobula (lo), as well as in the antennal lobe (D, H) and mushroom bodies (C, G). Scale bars: 200 mm (A-B, E-F) and 100 mm (C-D, G-H). Arrows indicate stained cell bodies.

In order to determine whether Apime-ASTA- and/or Apime-ASTC-receptors are likely to have a function in the brain, we studied the expression patterns of both receptor-encoding genes. *In situ* hybridization revealed specific but sparse staining throughout the brain for both receptor mRNAs (Fig 6). The distribution of cells expressing each type of allatostatin receptor gene appeared to be similar. In particular, staining was evident in the optic lobes, mostly in regions between the lobula, the medulla and the lamina (Fig 6B and 6F). Staining was also apparent in the mushroom bodies and particularly in Kenyon cells (mushroom body intrinsic neurons) (Fig 6C and 6G). Staining was also apparent in the antennal lobe region (Fig 6D and 6H) and in the sub-esophageal ganglion.

**Fig 7:**
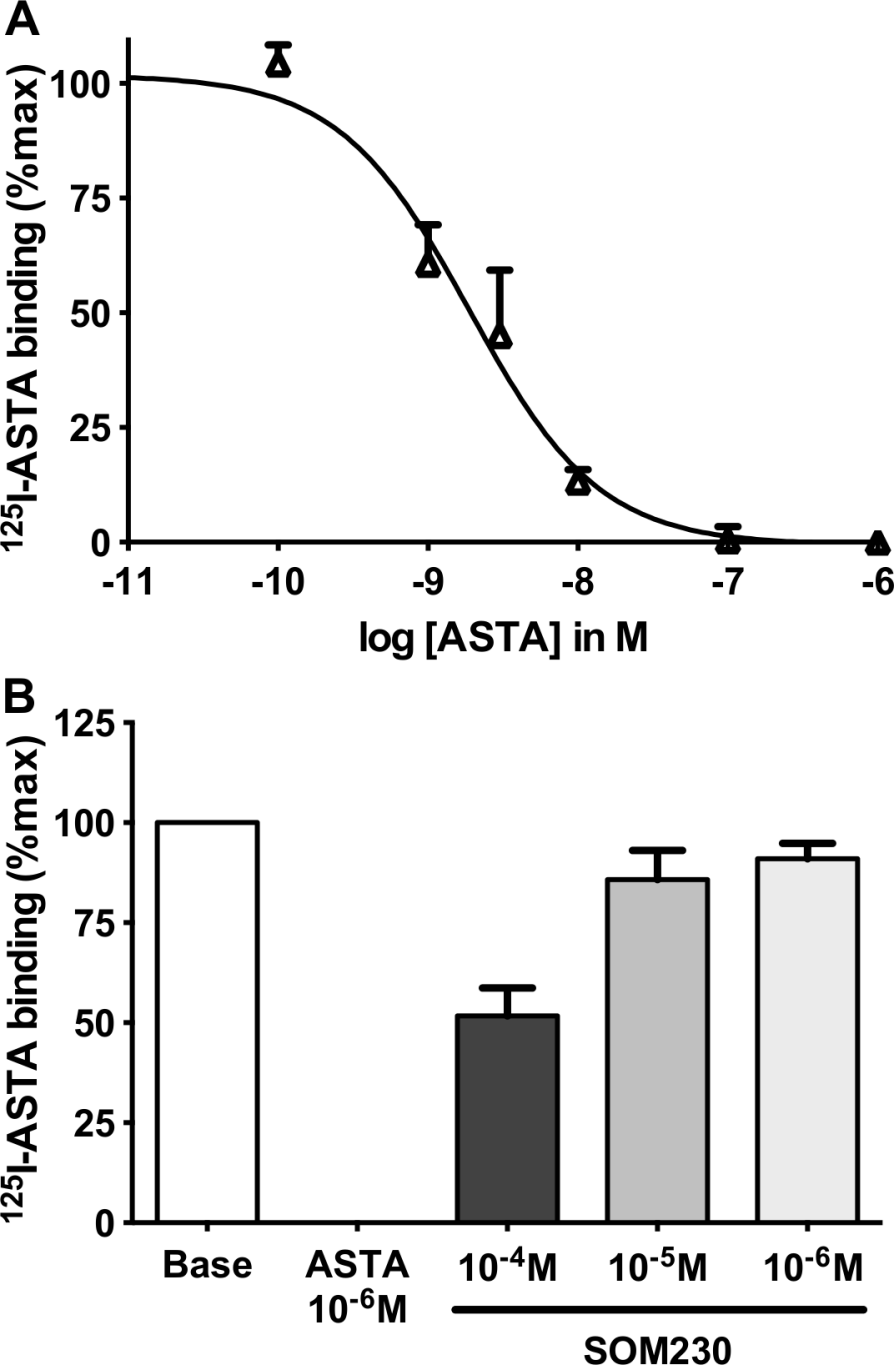
Binding of radiolabelled Apime-ASTA *in vivo* and *in vitro*. ^125^I-Apime-ASTA binding to bee brain crude membranes was displaced by increasing concentrations of non-labeled Apime-ASTA (A). Competitive binding with Apime-ASTA and increasing concentrations of a somatostatin homolog (SOM230) (B). Bars represent mean+/-SD (n = 3). Curves were obtained by fitting the data using nonlinear regression analysis in the GraphPad Prism software.

If, as expected from these observations, the receptor proteins are present *in vivo*, a functional correlate would be the existence of binding sites for allatostatins in the adult brain. We thus tested the ability of radiolabeled allatostatins to bind to cell membranes from crude brain extracts. Indeed, ^125^I-Apime-ASTA (technical limitations prevented us from testing Apime-ASTC and Apime-ASTCC) bound bee brain membranes and could be displaced by non-labeled ASTA in a dose dependent manner (IC_50_: 1.9 ± 0.3 nM, Fig 7A). Binding was specific since SOM230, a somatostatin analogue recognizing somatostatin receptors, inhibited binding with a very low affinity (IC_50_>100 μM) (Fig 7B).

### Allatostatin genes are expressed in the honey bee brain

**Fig 8:**
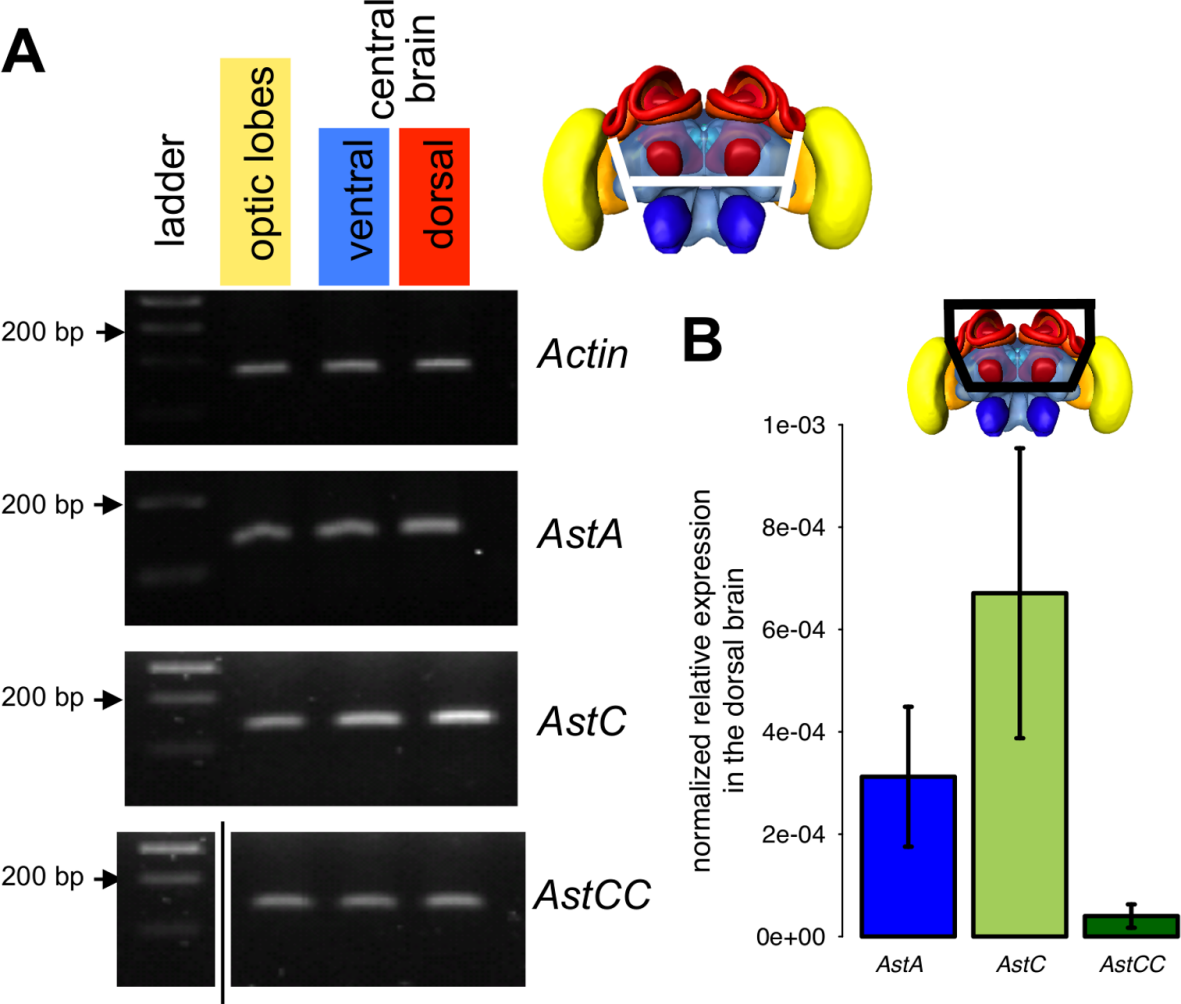
Gene expression of allatostatins. PCR was performed on cDNA samples obtained from different brain regions of adult worker bees: optic lobes (yellow in the bee brain illustration), ventral area (including antennal lobes in blue) and dorsal area (including mushroom bodies in red) of the central brain. (A) Electrophoresis on agarose gel of PCR fragments of *Actin* (used as a positive control 162 nt), *Apime-AstA* (120 bp), *Apime-AstC* (121 bp) and *Apime-AstCC* (105 bp, see S1 Table for primer sequences, original agarose gels are presented in S1 Fig). (B) qPCR was performed on dissected dorsal brain region from pollen foragers (n = 9, each sample run in triplicate) for the three allatostatin genes. Expression levels are normalized and relative to the geometric mean of *Am18S* and *elf1α*.

We examined also whether Apime-ASTC and Apime-ASTCC are expressed in the brain, as has been shown previously for Apime-ASTA [28]. We were able to detect expression of all three genes (*Apime-AstA*, *Apime-AstC* and *Apime-AstCC*) at the transcriptional level in the optic lobes, as well as in the ventral and dorsal halves of the central brain (containing respectively the antennal lobes and the mushroom bodies) (Fig 8A), thus suggesting widespread expression of these neuropeptides in the brain.

In addition, we did qPCR to determine the relative expression level of the three peptides, focusing on the dorsal part of the brain, which is likely to include the sources of allatostatins targeting the mushroom bodies (Fig 8B). We detected more *Apime-AstC* mRNA than that of *AstA*, and more so than *Apime-AstCC* mRNA. Furthermore, the individual variability was very high for these three genes (standard deviation close to 50%) but was stable for other genes, notably housekeepers (Am18S and elf1α, data not shown).

### Allatostatins modulate appetitive olfactory learning in a dose-dependent manner

**Fig 9:**
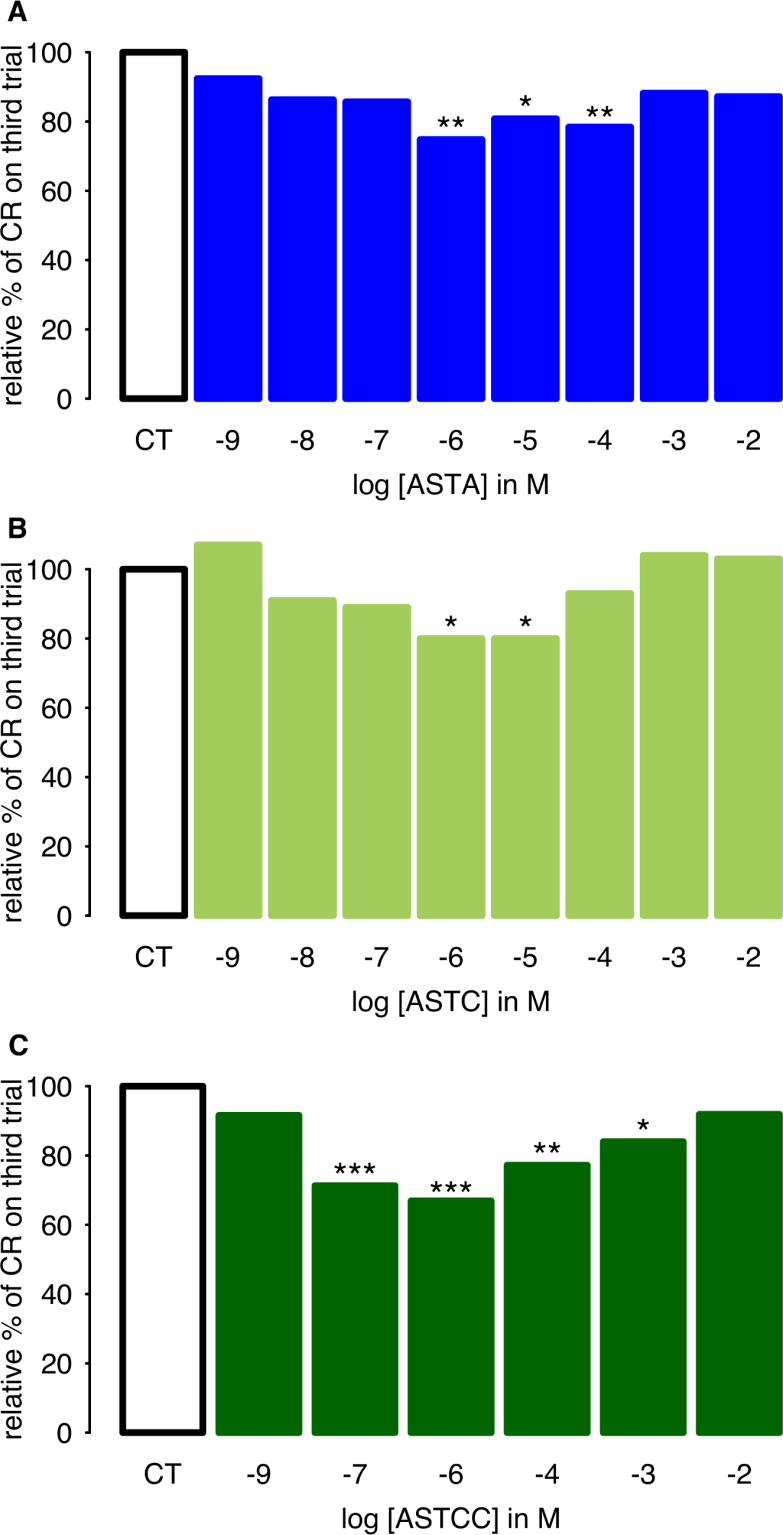
Dose-dependent learning impairment induced by allatostatins. Percentages of forager bees displaying conditioned proboscis extension responses (PER) in the third and last trial of conditioning, in honeybees injected with either vehicle alone (control bees, CT) or a given concentration of allatostatin A (A), C (B), or CC (C). For easier comparisons, **l**earning performances of control bees are set at 100% and those of all other groups are relative to CT. N = 120–182 (A), N = 70–80 (B) N = 56–87 (C). Significant decreases of learning performance, as compared to those of saline-injected bees, are indicated (*: p<0.05, **:p<0.01, ***:p<0.001).

We next asked whether allatostatins modulate olfactory learning in honey bees. Independent groups of bees were injected with different peptides prior to conditioning, and a range of concentrations of each peptide was tested. For all three of the peptides tested (Apime-ASTA, Apime-ASTC, Apime-ASTCC), allatostatin treatment affected the percentage of conditioned responses, as compared to vehicle-injected controls and the effects of each peptide were dose-dependent (Fig 9). Indeed, comparisons of performance in the last conditioning trial revealed significant decreases in learning performance in animals treated with doses in the micro-molar range of all three peptides (Fig 9A–9C): ASTA (GLM: 10^-4^M: -0.63±0.23, p<0.01; 10^-5^M: -0.57±0.23, p<0.05; 10^-6^M: -0.73±0.23, p<0.01), ASTC (10^-5^M: -0.84±0.42, p<0.05; 10^-6^M: -0.91±0.41, p<0.05) and ASTCC (10^-7^M: -1.59±0.44, p<0.001; 10^-6^M: -1.75±0.43, p<0.001; 10^-4^M: -1.35±0.44, p<0.01; 10^-3^M: -1.04±0.45, p<0.05). Interestingly, in all three cases, these effects followed U-shaped dose-response curves, with lower and higher doses having no effect on learning performance.

### Allatostatins do not alter responsiveness to odors or to sucrose

**Fig 10:**
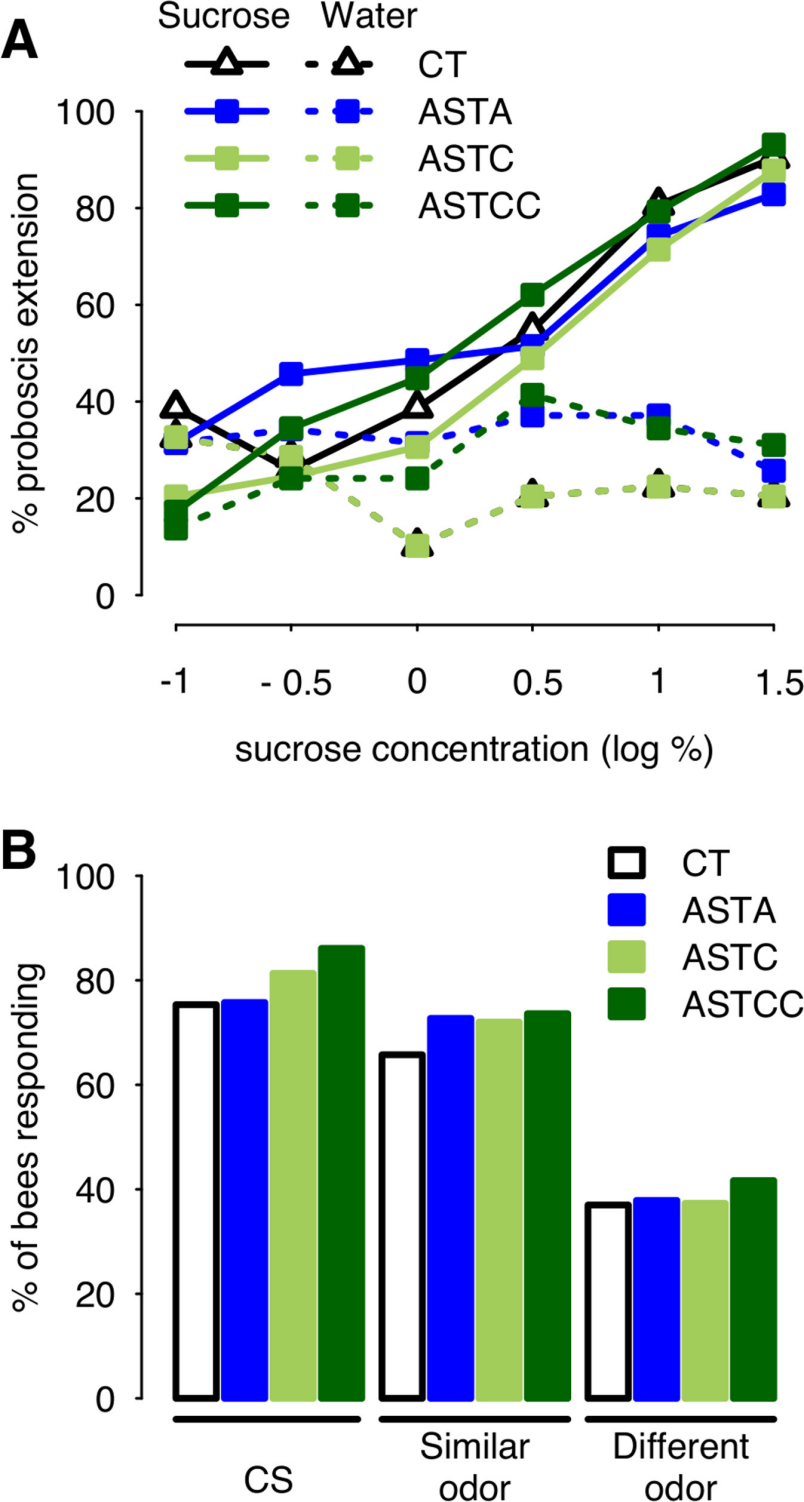
Intact response to sucrose and odors after allatostatin treatment. Responses elicited by stimulation of the antennae with increasing sucrose concentrations were tested following injection of saline (controls, *CT*) or either allatostatin (A). Overall, bees responded more to sucrose (solid line) than water (dashed line, GLM: 4.11±0.58, p<0.001), and the proportion of bees responding to sucrose increased with the concentration (GLM: 0.82±0.14, p<0.001) similarly in all groups, while the proportion of bees responding to water remained constant (GLM: -0.11±0.12, p = 0.34) (CT: N = 31, ASTA: N = 35 ASTC: N = 49, ASTCC: N = 29). Following olfactory appetitive conditioning, bees were tested for their capacity to generalize their learned responses to other odorants, as a way to assess their olfactory capacities (B). Allatostatin treatment had no impact on the response to any of the odors tested (CT: N = 73, ASTA: N = 66, ASTC: N = 75, ASTCC: N = 72). All bees were collected as they were leaving the hive, as for learning experiments.

Given reported effects of allatostatins on food intake and muscle activity in other species, it was important to examine the possibility that observed effects of allatostatins on associative olfactory learning in the bee might be an indirect consequence of changes in sucrose responsiveness (sensitivity to sucrose or motor control of the proboscis [64]). Sucrose sensitivity was tested after injection of Apime-ASTA, Apime-ASTC or Apime-ASTCC at 1μM, the lowest common effective concentration in the previous experiment. We found no significant effect of allatostatin treatment on the bees’ sensitivity to sucrose (GLM, ASTA: 0.22±0.72, p = 0.76; ASTC: 0.41±0.68, p = 0.55; ASTCC: 1.21±0.81, p = 0.13, Fig 10A) or water (ASTA: 0.53±0.69, p = 0.44; ASTC: -0.71±0.68, p = 0.30; ASTCC: 0.90±0.74, p = 0.22).

Possible impairment of olfactory discrimination was also tested for, using a generalization assay. Bees that have been trained to associate an odorant with a sucrose reward can generalize their response to other, new odorants, and their degree of generalization accounts for the perceptual similarity between each odorant and the conditioned stimulus [66]. Thus, we asked whether injection with either allatostatin (1μM) after learning might affect such ability. Irrespective of their treatment, bees responded more to the CS than to an odorant similar to the CS, and more to a similar than to a dissimilar odorant (Fig 10B). Allatostatin treatment had no impact on the response to any of the odors tested (1-nonanol: p>0.1 for all three peptides [ASTA 0.02±0.40, p = 0.96; ASTC 0.35±0.40, p = 0.37; ASTCC 0.71±0.44, p = 0.10]; nonanal; p>0.3 [ASTA 0.33±0.37, p = 0.38; ASTC 0.29±0.36, p = 0.41; ASTCC 0.37±0.36, p = 0.30]; 1-hexanol: p>0.5 [ASTA 0.04±0.35, p = 0.91; ASTC 0.01±0.34, p = 0.96; ASTCC 0.20±0.34, p = 0.56]). Thus, ASTs did not impair olfactory responses, though injected at a dose that had a significant impact on olfactory learning.

Taken together, these results suggest that ASTs act on learning mechanisms *per se* rather than on sensory or motor processes required for the expression of learning.

## Discussion

The presence of allatostatins in the brain of adult insects has led researchers to suggest that these neuropeptides may modulate the function of neural circuits in the brain [23–24, 28,75–77]. The results of our study support this hypothesis. Apime-ASTA, Apime-ASTC and Apime-ASTCC, together with receptors that mediate the actions of these peptides (Apime-ASTA-R and Apime-ASTC-R, respectively) appear to be widely expressed in the brain of the adult worker honey bee, including in the optic lobes, antennal lobes and mushroom body region, as well as in the subesophageal ganglion.

When expressed *in vitro*, Apime-ASTA- and Apime-ASTC-receptors responded selectively to A-type and C-type peptides, respectively, with no detectable cross-activation. Both ASTC and ASTCC activated the C-type receptor in the honey bee, as was observed in *Tribolium castaneum* [78]. Consistent with our prediction that both receptors would be likely to couple to G*α*_*i/o*_ and to inhibit the production of cAMP, we found that expressing either Apime-ASTA-R or Apime-ASTC-R in HEK293 cells reduced intracellular cAMP to a level significantly lower than that detected in mock-transfected cells. This inhibitory activity was agonist-independent, a feature that is characteristic of a significant proportion of mammalian and insect GPCRs [79–81], provided the activity levels are compared to mock cells rather than non-stimulated cells transfected with the studied receptor. For example, others [58] have shown reduced bioluminescence after expressing the D2-like dopamine receptor TricaDop3 in HEK cells in absence of dopamine. Similarly, expressing the fly ASTA receptor in Chinese hamster ovary cells induced a high GTPγS activity in absence of ligand [82], suggesting an agonist-independent constitutively active state of the receptor. The fact that calcium measurements did not show evidence of constitutive activity of the receptors is not contradictory. Indeed, GPCRs are able to couple with more than one G protein, and constitutive activity can selectively involve one signaling pathway (see [83] for a review).

Interestingly, in the case of Apime-ASTA-R, this constitutive inhibitory activity could be overcome in a dose-dependent manner by applying ASTA to Apime-ASTA-R-expressing cells: luciferase activity increased, reaching values close to those detected in mock cells (and even higher, in one experiment). In this case, A-type peptide appeared to function as an inverse agonist increasing cAMP in Apime-ASTA-R-expressing cells to a level significantly above that detected in mock-transfected cells. Although uncommon, endogenous inverse agonists have been described previously [84–85]. Inverse agonists typically reverse constitutive activity, sometimes beyond a mere return to basal levels [86], as was observed in one transfection when Apime-ASTA-R-transfected cells were stimulated with ASTA (Fig 5C). Two mechanisms are possible: binding of ASTA could potentially decrease the coupling between Apime-ASTA-R and the G*α*_*i/o*_ protein, a mechanism described elsewhere for the cannabinoid receptor [86–87], or could shift the coupling of the receptor to a different G protein; this has been proposed for Drome-ASTA-R [82]. Further investigation is needed to better understand the coupling of this receptor.

The constitutive inhibitory activity associated with the expression of Apime-ASTC receptors in HEK293 cells could not be overcome in the same way. While application of high concentrations of ASTCC to Apime-ASTC-R expressing cells increased cAMP levels in the cells slightly, this occurred also in mock-transfected cells, suggesting that ASTCC activates a population of receptors endogenous to HEK293 cells. ASTCC may activate somatostatin receptors, which are expressed by HEK293 cells [88] and, as our phylogenetic analyses reveal, are more closely related to Apime-ASTC-R than to Apime-ASTA-R. In contrast to ASTCC, ASTC failed to increase intracellular cAMP levels in cells expressing ApimeASTC-R, or in mock-transfected cells.

While further work is required to confirm the functional properties of Apime-ASTC and Apime-ASTA receptors, the relationship between C and CC allatostatins deserves particular mention. Contrary to the situation in Dipterans [50,89], in the honey bee genome there appears to be only one gene that encodes a receptor of the ASTC-type ([29], present investigation), a situation similar to that reported for the genome of the red flour beetle, *Tribolium castaneum* [90]. Strikingly, in the present investigation, both peptides (Apime-ASTC and Apime-ASTCC) activated Apime-ASTC-R with very similar EC_50_ values. While this lends support to the view that the two peptides may be structurally very similar [19], at the behavioral level we found that effects of ASTCC were apparent at much lower doses than effects of ASTC. This suggests that *in vivo*, Apime-ASTC receptors may have a higher affinity for Apime-ASTCC than for Apime-ASTC. The relative abundance of these two peptides in the honey bee brain may also differ. Consistent with the situation in *D*. *melanogaster* [19], our study suggests that Apime-ASTCC is likely to be present at lower levels than Apime-ASTC, at least in the mushroom body region. This might explain the detection of ASTC only (identified as LRNQLDIGDLQ) in an earlier study on honey bee foragers based on quantitative peptidomics [91]. To our knowledge, the ASTCC peptide has never been detected in any invertebrate species [91–94], so its role as a bioactive peptide remains putative. As worker honey bees undergo significant physiological and behavioral changes during their adult lifetime, allatostatin signaling in these insects might be age- or behavior-dependent, as suggested recently for ASTA in Africanized honey bees [95]. We are currently exploring this interesting possibility using a comparative approach to examine different behavioral castes.

As our phylogenetic analysis confirms, Apime-ASTA-R and Apime-ASTC-R belong to two distinct receptor families that include, respectively, mammalian galanin and somatostatin receptors [19,50,96–98]. Yet, as is often the case for small neuropeptides, this conservation does not fully apply to the ligands. Indeed, Apime-ASTA bears no resemblance to mammalian galanin but Apime-ASTC shares some similarity with somatostatin [96–97]. Despite such divergence, the functions of galanin/ASTA and somatostatin/ASTC/ASTCC pathways appear to have been conserved during evolution. All share a predominantly inhibitory action in common. For example, somatostatins were primarily identified as inhibiting the secretion of growth hormone [99], and for their action as inhibitory hormones in the gastro-intestinal tract [100], two functions shared by many insect allatostatins [101]. Similarly, galanin, despite its stimulatory action on food intake, has many inhibitory effects, notably on osmotic regulation, nociception, and more generally on neuron excitability [102–103]. Interestingly, both galanin and somatostatin modulate learning mechanisms and, when injected, can impair learning performance [104–105] through the modulation of synaptic plasticity [106–108]. These peptides and their receptors are expressed in regions of the mammalian brain that sustain learning-dependent plasticity (e.g. hippocampus) [3] and indeed, the suggestion that allatostatins might play a similar role in insects arose from the identification of these neuropeptides in the mushroom body region [28,95,109], prominent structures known to play a critical role in learning and memory processes in insects [31]. All three allatostatin-encoding genes in the honey bee appear to be expressed in this region of the brain, and we show here that their putative receptors are transcriptionally expressed also by mushroom body intrinsic neurons (Kenyon cells). As a possible functional correlate, we tested the ability of allatostatins to modulate learning in the bee, and found that increasing the titer of any of one of the three peptides (ASTA, ASTC or ASTCC) in the head capsule led to a significant, dose-dependent (yet not linear) decrease in the percentage of bees that learned to associate an odorant with a sucrose reward. Treatment with allatostatin had no effect on olfactory discrimination, suggesting that the neuropeptides affect associative olfactory learning processes rather than olfaction *per se*.

Accordingly, our *in situ* hybridization data shows that the two receptor genes are expressed in cell bodies of mushroom body intrinsic neurons (Kenyon cells). Because a previous study could not reliably detect allatostatins honey bee MB calyces [93], we believe that our detection of allatostatin mRNAs in the dorsal brain is most likely due to expression outside of the MBs. This suggests possible modulation of MB activity from extrinsic neurons, such as those ramifying into the MB calyces and vertical lobes as described by [28] in their study of ASTA distribution (the only one available for the bee brain). Similarly, our observation of receptor mRNA within the antennal lobes is consistent with their innervation by ASTA-positive neurons [28] and with modulation of olfactory learning. It should be noted that we cannot exclude, at this point, that some of the cells producing allatostatins may be glial cells, as suggested earlier in *Drosophila* [50]. All in all, the expression pattern of Apime-ASTA-R mRNA as revealed by our *in situ* hybridization is consistent with the expression of the peptide. Indeed, similarly to the MBs and ALs, abundant allatostatinergic innervation was described in the other main regions where the receptor mRNA is found (optic lobes, subesophageal ganglion) [28]. This is consistent with a local action of ASTA, but does not exclude a possible modulation through neuroendocrine signaling, as suggested by their behavioral effects when injected in the head haemolymph.

In cockroaches A-type allatostatins regulate feeding [110], and in fruit flies this function is under the control of ASTA-expressing cells [20], which also influence foraging behavior and metabolism [23–24]. This might be the case in honey bees also as ASTA is abundant in the subesophageal ganglion of the bee ([28], present investigation), a region involved in regulating food intake and controlling proboscis extension, which is triggered reflexively by sucrose [111–112]. While transcripts for Apime-ASTA- and Apime-ASTC-receptors were detected consistently in this region, we found nonetheless that bees injected with 1μM of ASTA, ASTC or ASTCC responded as well to 50% sucrose stimulations as control bees. Bees treated with these peptides (at a concentration of 1μM) also seemed to feed normally and displayed sucrose responsiveness that was not significantly different from those of controls. Although we cannot discard the possibility that other doses might affect motivation for sucrose, clearly changes in sucrose sensitivity cannot account for the appetitive learning deficits evident in this study. Thus, taken together our data suggest a function for allatostatins as modulators of learning in the bee. To our knowledge, this is the first evidence of allatostatin function in the adult honey bee brain. How this is achieved remains to be determined, but immunohistochemistry data [28,109] suggest that modulation of neural circuits involved in learning may be a function common to several insect species. Thus, we propose that the involvement of allatostatin in learning modulation may represent a conserved function, the ecological significance of which remains to be explored.

## Supporting Information

S1 Fig Electrophoresis on agarose gel of PCR fragments of *Actin* (used as a positive control 162 nt, A), *Apime-AstA* (120 bp, B), *Apime-AstC* (121 bp, C left) and *Apime-AstCC* (105 bp, C right). (TIFF)

S1 Table Primers for RT-PCR. F: forward, R: reverse.
(TIFF)

S2 Table Primers for qPCR. RE: reaction efficiency; MP: product melting point, F: forward, R: reverse.
(TIFF)
