# Supplementary figures and images for: Honey Bee Allatostatins Target Galanin/Somatostatin-Like Receptors and Modulate Learning: A Conserved Function?
Source: PLoS One. 2016 Jan 7;11(1):e0146248. doi: 10.1371/journal.pone.0146248 (PMC4704819; doi:10.1371/journal.pone.0146248)

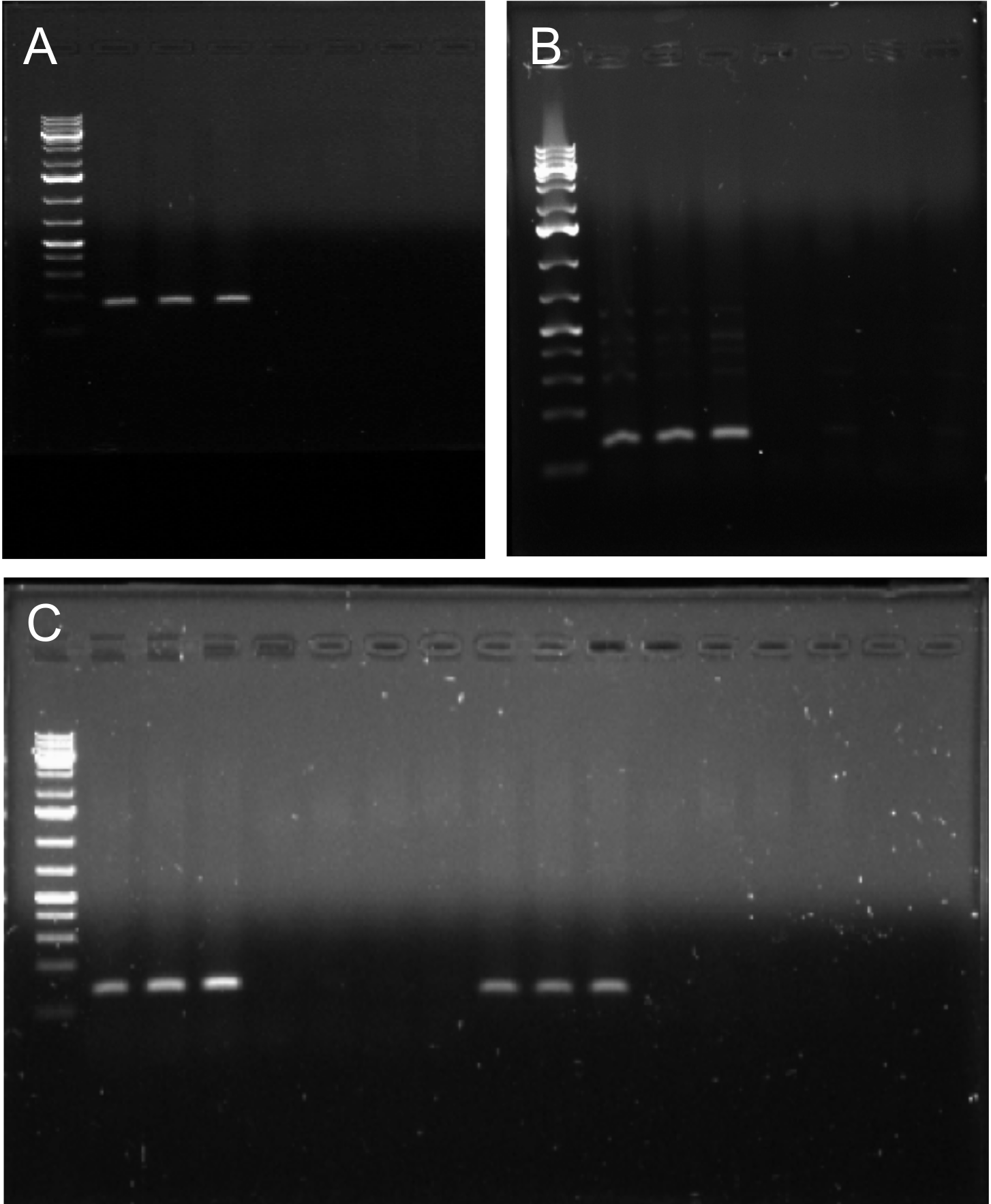

Supplement: S1 Fig — (TIFF) [file pone.0146248.s001.tiff]

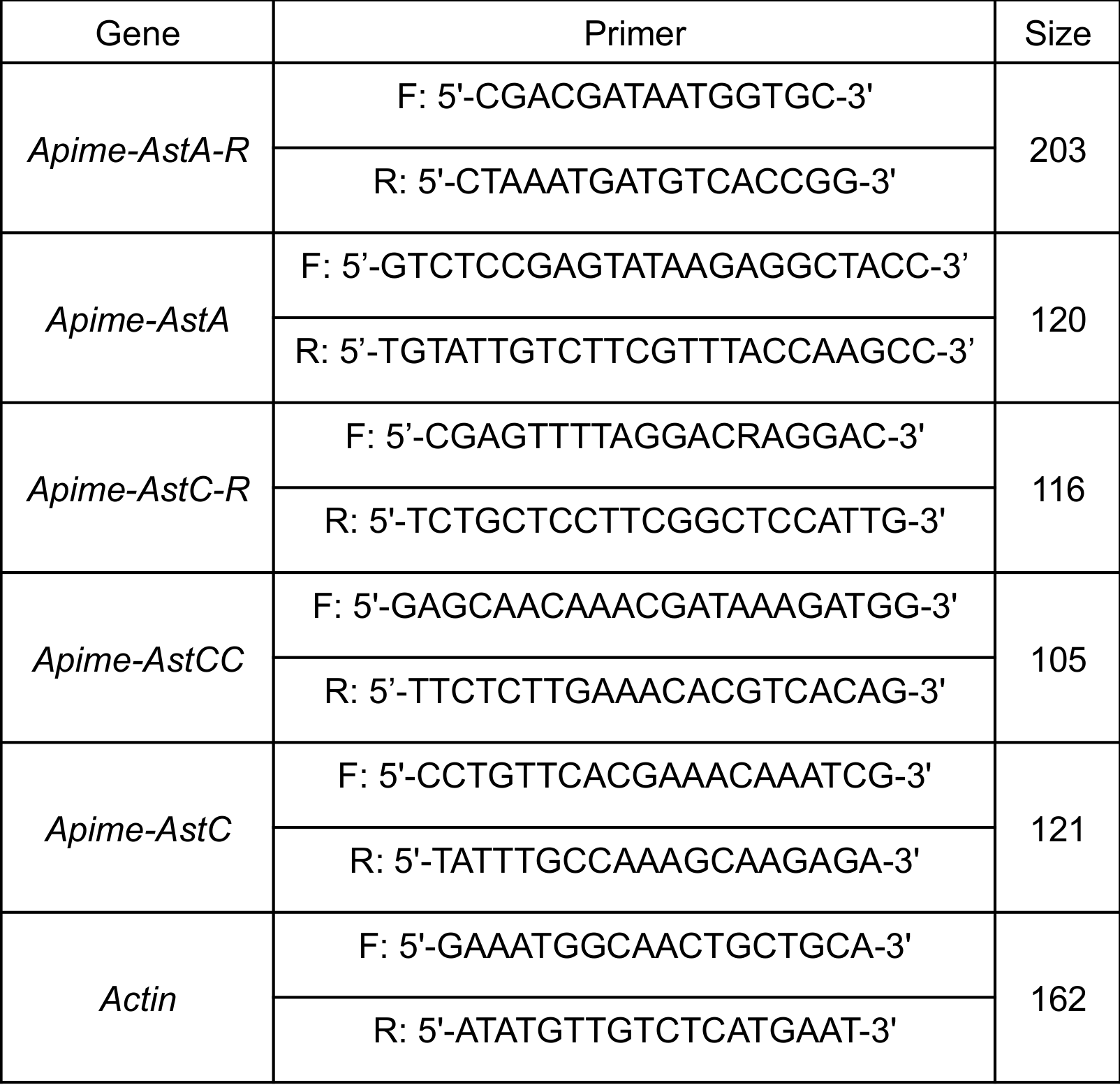

Supplement: S1 Table — F: forward, R: reverse. (TIFF) [file pone.0146248.s002.tiff]

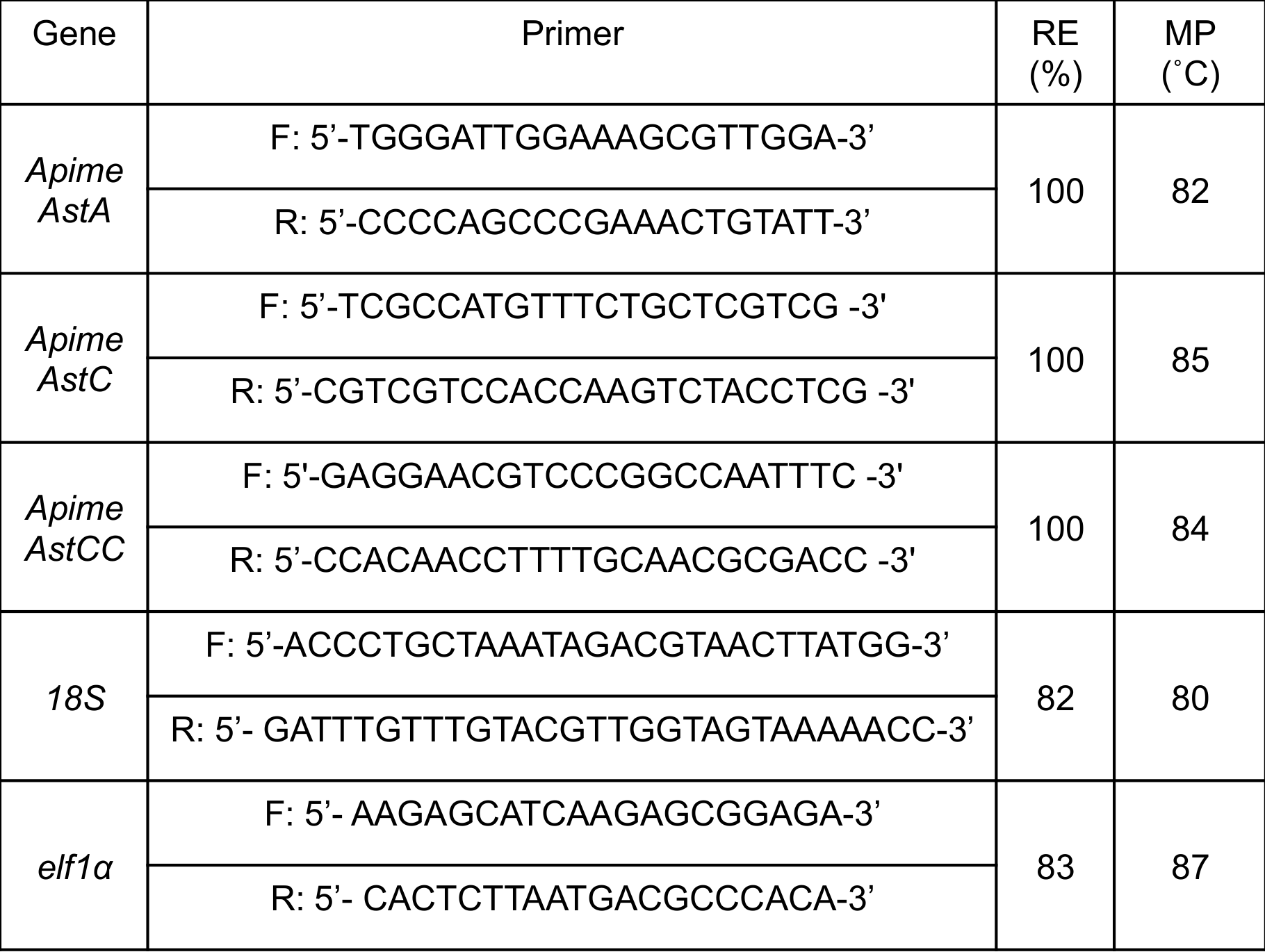

Supplement: S2 Table — RE: reaction efficiency; MP: product melting point, F: forward, R: reverse. (TIFF) [file pone.0146248.s003.tiff]
